# Supplementary material for: Living the employer brand during a crisis? A qualitative study on internal employer branding in times of the COVID-19 pandemic
Source: PLoS One. 2024 May 13;19(5):e0303361. doi: 10.1371/journal.pone.0303361 (PMC11090342; doi:10.1371/journal.pone.0303361)
Supplement: S1 Table — (DOCX) [file pone.0303361.s001.docx]

S1 Table. Demographic information on the cases and respondents of the interviews, member check, and employee check (N = 37 + 6 + 6)

| **#** | **Size of the organization** | **Sector** | **Gender** | **Tenure**  **(years)** | **Age**  **(years)** |
| --- | --- | --- | --- | --- | --- |
| **IN_1^a^** | 1500 employees | Private: Agriculture, Food,  and Natural Resources | Female | 2 | 38 |
| **IN_2** | 900 employees | Public: Medicine | Female | 13 | 58 |
| **IN_3** | 1530 employees | Private: Finance | Female | 3 | 25 |
| **IN_4** | 575 employees | Private: Agriculture, Food,  and Natural Resources | Male | 4 | 54 |
| **IN_5** | 1200 employees | Private: Social Sciences | Male | 15 | 52 |
| **IN_6** | 2300 employees | Private: Manufacturing | Male | 31 | 51 |
| **IN_7** | 5200 employees | Private: Science, Technology, Engineering  and Mathematics | Male | 4.5 | 51 |
| **IN_8** | 325 employees | Private: Information  Technology | Male | 14 | 35 |
| **IN_9** | 87 employees | Private: Information  Technology | Female | 0.5 | 54 |
| **IN_10** | 140 employees | Private: Architecture and  Construction | Female | 1.5 | 53 |
| **IN_11** | 120 employees | Private: Information  Technology | Female | 26 | 52 |
| **IN_12** | 1400 employees | Private: Transportation,  Distribution, and Logistics | Female | 16 | 41 |
| **IN_13** | 185 employees | Private: Transportation,  Distribution and Logistics | Female | 12 | 45 |
| **IN_14** | 120 employees | Private: Private: Agriculture, Food, and  Natural Resources | Female | 7 | 36 |
| **IN_15** | 73 employees | Private: Business  Management and Administration | Female | 20 | 46 |
| **IN_16** | 1540 employees | Private: Finance | Female | 30 | 62 |
| **IN_17** | 285 employees | Private: Information Technology | Male | 4 | 27 |
| **IN_18** | 4000 employees | Private: Social Sciences | Male | 11 | 60 |
| **IN_19** | 110 employees | Private: Manufacturing | Female | 3 | 53 |
| **IN_20** | 3500 employees | Private: Science, Technology, Engineering and Mathematics | Male | 5 | 45 |
| **IN_21** | 3000 employees | Private: Transportation, Distribution and Logistics | Female | 12 | 36 |
| **IN_22** | 650 employees | Public: Government & Public Administration | Male | 27 | 51 |
| **IN_23** | 1500 employees | Private: Transportation, Distribution and Logistics | Female | 9 | 31 |
| **IN_24** | 100 employees | Private: Finance | Male | 30 | 54 |
| **IN_25** | 43 employees | Private: Finance | Female | 5 | 38 |
| **IN_26** | 65 employees | Private: Manufacturing | Female | 5 | 45 |
| **IN_27** | 40 employees | Private: Information  Technology | Male | 30 | 52 |
| **IN_28** | 34 employees | Private: Finance | Female | 20 | 54 |
| **IN_29** | 43 employees | Private: Information  Technology | Female | 1.5 | 32 |
| **IN_30** | 65 employees | Private: Finance | Female | 6 | 32 |
| **IN_31** | 45 employees | Private: Business  Management and  Administration | Male | 21 | 45 |
| **IN_32** | 22 employees | Private: Information  Technology | Female | 3 | 50 |
| **IN_33** | 400 employees | Private: Information  Technology | Male | 21 | 46 |
| **IN_34** | 35 employees | Private: Transportation,  Distribution and Logistics | Female | 5 | 30 |
| **IN_35** | 48 employees | Private: Information  Technology | Male | 21 | 44 |
| **IN_36** | 35 employees | Private: Information Technology | Male | 3 | 51 |
| **IN_37** | 50 000 employees | Private: Information Technology | Female | 6 | 45 |
| **MC_1** | 31 employees | Private: Business Management and Administration | Female | 31 | 52 |
| **MC_2** | 2100 employees | Public: Education and Training | Female | 2 | 51 |
| **MC_3** | 1000 employees | Public: Medicine | Male | 8 | 31 |
| **MC_4** | 4000 employees | Public: Education and Training | Female | 6 | 38 |
| **MC_5** | 1300 employees | Public: Medicine | Female | 6 | 31 |
| **MC_6** | 11 000  employees | Private: Retail | Female | 4 | 34 |
| **EC_1** | 1530 employees | Private: Finance | Female | 7 | 30 |
| **EC_2** | 120 employees | Private: Private: Agriculture, Food, and Natural Resources | Female | 22 | 58 |
| **EC_3** | 65 employees | Private: Manufacturing | Female | 3 | 35 |
| **EC_4** | 185 employees | Private: Transportation, Distribution, and Logistics | Female | 8 | 64 |
| **EC_5** | 325 employees | Private: Information Technology | Female | 5 | 47 |
| **EC_6** | 325 employees | Private: Information Technology | Male | 3 | 28 |
| ^a^IN refers to the respondents of the interviews. MC refers to the respondents of the member check. EC refers to the respondents of the employee check. | | | | | |
