# Supplementary material for: Living the employer brand during a crisis? A qualitative study on internal employer branding in times of the COVID-19 pandemic
Source: PLoS One. 2024 May 13;19(5):e0303361. doi: 10.1371/journal.pone.0303361 (PMC11090342; doi:10.1371/journal.pone.0303361)
Supplement: S2 Table — (DOCX) [file pone.0303361.s002.docx]

**S2 Table. Minimal dataset including data availability statement, ethical elements, and data-analysis process.**

| Title | Qualitative Data Sharing Covid-19 Crisis: Participant Understanding, Consent forms, Excerpts of relevant parts, Dataset member check and employee check |
| --- | --- |
| Description: a summary describing the purpose, nature, and scope of the data project | **Description:** This project focuses on a qualitative exploration of sustainable HR practices amid the COVID-19 pandemic, with a specific emphasis on internal employer branding processes. The research team employed semi-structured interviews to delve into the experiences and perspectives of HR managers regarding internal employer branding during these unprecedented times.  **Project Summary:** The primary objective of this project is to understand the nuances of internal employer branding in the context of the COVID-19 pandemic. Through semi-structured interviews conducted with 37 HR managers (*sample 1*), the research team aimed to capture diverse insights and experiences related to employer branding processes within organizations. To enhance the credibility and validity of the findings, a member check was conducted. This involved reaching out to a subset of participants (6) (*sample 2*), presenting them with summarized research findings, and seeking validation or clarification. This iterative process ensures that the interpretations align with participants' actual experiences and perspectives. This project contributes to the broader understanding of how HR practices, specifically internal employer branding, evolved during the unique challenges posed by the COVID-19 pandemic. The utilization of qualitative methods provides depth and context to the experiences of HR managers, shedding light on the dynamic landscape of organizational practices during this critical period. Lastly, to verify and compare the intended HR managers initiatives to the perceived, experienced impact on employees, we interviewed six employees from the respectively organizations (*sample 3*), who took part in the interviews (sample 1). We compared the results of the 37 interviews HR managers with the perceptions and experiences of the employees.  **Data overview:** The data consist of excerpts from semi-structured interviews with 37 different HR managers during January and February of 2020. The member check, involved reaching out to a subset of participants (6). By presenting them with a summarized version of the research findings, the research team sought validation and additional insights. This iterative process ensures that the interpretations align closely with the participants' actual experiences and perspectives, adding an extra layer of rigor to the research outcomes. We enclosed the results of the member check in Table 1 of the manuscript. Furthermore, we conducted an employee check, interviewing employees based on the insights gained from discussions with HR managers to inquire about their experiences. The data for the employee check is included in the S4_Tab, however, recordings and the personal notes are not enclosed. However, notes are available upon request. Please note that the recordings cannot be shared. |
| Subject | Social sciences |
| Keywords | Qualitative research, semi-structured interviews, thematic analysis, exploration analysis, member check, and employee check |
| Related publication | Not yet published (in revision round for publication Plos One) |
| Time period | Interviews with HR manager & member check: December 2020 – April 2021 + Interviews employees: December- |
| Date of data collection | Interviews with HR manager & member check: December 2020 – April 2021 + Interviews employees: December- |
| Geographic coverage | Organizations in Flanders, Belgium |
| Language | Dutch |
| Funding information | No funding received for this study |
| Data availability statement: | Excerpts from transcripts of HR managers/CEO’s relevant to this study are accessible as Supporting information (see Supporting information ‘S5_Tab’). Readers can refer to this material for a deeper understanding of the analyzed qualitative data. To uphold confidentiality and adhere to participant consent, these excerpts are presented in a manner that aligns with the agreed-upon terms. Regrettably, complete interviews cannot be provided, as participants have not authorized the publication of entire transcripts.  The data supporting the findings of the member check are accessible within this article (see Table 1) . The complete set of survey questions used for the member check stage can be found below (see “Member check questions”). Additionally, participant demographic data and member check results are presented in Supporting information (S1_Tab and S3_Tab). The anonymized datasets utilized and/or analyzed during the current study are available from the corresponding author upon request. The Qualtrics raw datafile for the member check is also available from the corresponding author upon request. Participants did not explicitly grant permission for the public sharing of this information.  The data for the employee check, including recordings and personal notes, is not enclosed. However, notes are available upon request. Please note that the recordings cannot be shared. Interview questions used for the employee check can be found below (see “employee check questions”). Additionally, participant demographic data are presented in Supporting information (S1_Tab).  All other data supporting the findings of this study are securely stored on password-protected servers hosted by Ghent University. Digital recordings, full interview transcripts, and related documents are anonymized and coded to ensure participant confidentiality. Access to the stored data is restricted to the primary researchers involved in the project, including the principal investigator. Access is granted solely for the purpose of data analysis, and strict confidentiality agreements must be adhered to by authorized researchers. No identifiable information is disclosed at any stage of data sharing. |
| Consent form example | **Informed consent form:**  ***a) Consent form interviews HR managers***  I, the undersigned ………………………………………………….. (name of the respondent), state hereby that:  (1) I have received sufficient information about this study and have been given the opportunity to ask additional questions.  (2) I am voluntarily participating in this research.  (3) I have been informed of the option to discontinue the cooperation with this research at any time without further obligations.  (4) I have been informed that the interview will be recorded and will be treated confidentially.  (5) I give permission to the researchers of Ghent University to store my answers anonymously, to process them and to report them for scientific purposes.  (6) I have been informed of the possibility to request a general summary of the research findings afterwards.  Read and approved in …………………………………. (location) on ……………………..………. (date)  Name + Signature  ***b) Consent form: member check***  By participating in this study, you are agreeing to the following conditions:   1. Please reply honestly. There are no right or wrong answers.   2. Any responses you provide will be anonymized so that neither the research team nor additional respondents will know which answers are from you.   3. By partaking in this study, you agree that the information we collect during this study will be used (solely) for scientific purposes.   4. You have the opportunity to terminate this survey whenever you like.    By selecting "I agree", you are consenting to the conditions described above.  ***c) Consent form: interviews employees***  I, the undersigned ………………………………………………….. (name of the respondent), state hereby that:  (1) I have received sufficient information about this study and have been given the opportunity to ask additional questions.  (2) I am voluntarily participating in this research.  (3) I have been informed of the option to discontinue the cooperation with this research at any time without further obligations.  (4) I have been informed that the interview will be recorded and will be treated confidentially.  (5) I give permission to the researchers of Ghent University to store my answers anonymously, to process them, and to report them for scientific purposes. I understand that while parts of the interview may be shared for scientific purposes, non-public sharing will be maintained.  (6) I have been informed of the possibility to request a general summary of the research findings afterwards.  Read and approved in …………………………………. (location) on ……………………..………. (date)  Name + Signature |
| Interview questions (sample 1) | ***Interview questions examples:***  1. What do you understand by internal employer branding?  2. Do you undertake actions related to internal employer branding?  a. If yes, what are they?  3. How does your internal employer branding differ from pre-COVID-19 times?  4. Has the (internal) communication method changed due to this?  a. If yes, in what way?  b. Are you now using more digital technology/tools such as intranet, newsletters, ...?  c. Will you continue to use these tools after the COVID-19 period?  5. What were the major challenges around internal employer branding before COVID-19 (and how do these differ from the challenges you are currently facing?)  6. How do you ensure that your employees stay loyal and committed during COVID-19?  a. Does this differ from pre-COVID-19? Have you noticed more or less commitment now?  ***Adapted interview questions:***  1. What do you understand by internal employer branding? (If necessary, provide a definition). nternal Communication  2. How did your internal communication to employees take place before COVID-19?  a. What concrete actions did you take to convey the organization's 'DNA' to employees?  b. What effects did this internal communication have on the employee?  3. How is your internal communication now, in times of COVID-19, towards employees?  a. Are you now using more digital technology/tools such as intranet, newsletters,...?  b. What influence does digital communication have on employees?  c. What influence does digital communication have on the connectedness employees still have?  d. How do you measure or observe these effects?  e. What is the effect of COVID-19 on face-to-face communication?  4. What challenges has COVID-19 posed for your internal communication?  a. What effect did this have on the organization and on your internal employer brand?  5. Influence of Leadership: How do you ensure that the leader conveys the organization's values to his employees in the period before COVID-19?  a. What concrete actions did the leader take in this regard?  b. What was your (manager/HR department) role in this?  c. What effect did the leader have on employees as a result?  6. How do leaders promote engagement in the organization before COVID-19?  a. How has the role of the leader changed regarding internal employer branding during the times of COVID-19?  b. What effect does this have on the employee? How is this measured/observed?  7. What challenges regarding leadership have come to light during COVID-19?  a. How did this manifest in the organization?  8. Employee-Organization Relationship (EOR): What concrete actions did you take to promote a good relationship between the employee and the organization before COVID-19?  a. What effect did this have on the employee, e.g., more engagement, more ambassadorship?  b. How did you notice this? How was this measured?  9. What challenges or difficulties do you face in times of COVID-19 regarding binding the employee to your organization?  a. What is the effect on your internal employer branding?  b. If negative, how and with what concrete actions have you tried to counteract this?  10. What effect did COVID-19 have on the relationship between the employee and the organization? For example, less engagement? Less doing extra things for the organization? |
| Member check questions (sample 2) | 1. HR managers had the feeling that employees had more difficulties connecting with the internal employer brand during the COVID-19 pandemic. Did you have the same feeling for your organization?   - Would you like to comment on this statement?   2. Due to the COVID-19 pandemic, HR managers were encouraged to reflect on the content of their internal employer brand. Some HR managers mentioned that the COVID-19 pandemic was an opportunity to improve their employer brand.  Did you have the same feeling for your organization?   - Would you like to comment on this statement?   3. During the pandemic, HR managers reported that they continued to focus on internal employer branding, sometimes even more than before the pandemic. Did you have the same feeling for your organization?   - Would you like to comment on this statement?   4. For some organizations, the internal aspect of employer branding (= managing the employer brand among current employees) was more important during the pandemic than the external aspect of employer branding (= managing and promoting the employer brand among potential employees). Did you have the same feeling for your organization?   - Would you like to comment on this statement?   5. HR managers had the feeling that it was more difficult to transfer the employer brand among new employees during the COVID-19 pandemic as they have less knowledge and experience with the internal employer brand.  Did you have the same feeling for your organization?   - Would you like to comment on this statement?   6. According to HR managers, the feeling of a common enemy (enemy = COVID-19 pandemic) encouraged employees to connect with the employer brand. Did you have the same feeling for your organization?   - Would you like to comment on this statement?   7. During the COVID-19 pandemic, the old methods or strategies to communicate the employer brand among current employees were not always possible. Did you have the same feeling for your organization?   - Would you like to comment on this statement?   8. HR managers reported that they had to search for new methods to transfer and communicate the employer brand among current employees. Did you have the same feeling for your organization?   - Would you like to comment on this statement?   9. HR managers reported that because of the COVID-19 pandemic the communication style or how the employer brand is communicated to employees was now more centered on expressing warmth and care. Did you have the same feeling for  your organization?   - Would you like to comment on this statement?   10. HR managers noticed that during the COVID-19 pandemic they did not receive or received less bottom-up communication about the employer brand. As a result of the lack of bottom-up communication and/or interpersonal communication, some  HR managers reported that they did not know how the employer brand communication was received and that there may have been misinterpretations or multiple forms of the employer brand during the COVID-19 pandemic.  Did you have the same feeling for your organization?   - Would you like to comment on this statement?   11. Due to the COVID-19 pandemic, HR managers had the feeling that employees were overflowed with a lot of different forms of communication (COVID-19 regulations, corporate communication, operational communication, etc.). As a result, HR  managers had the feeling that employer branding communication was difficult to differentiate from other communication or could stand out from other communication. Did you have the same feeling for your organization?   - Would you like to comment on this statement?   12. Some HR managers reported that they installed “target group communication management” during the COVID-19 pandemic. The purpose of this practice was to only communicate what was necessary for certain groups of employees.  As a result, HR managers believed this facilitated employees to differentiate the employer brand communication from other communication and better understand and implement it in their daily jobs. Did you have the same feeling for your organization?   - Would you like to comment on this statement?   13. HR managers noticed that during the pandemic supervisors played a key role in transferring and radiating the employer brand to their employees. Their role seemed to become even more important than before the pandemic (as they were often  the main contact of the employee with the organization). Did you have the same feeling for your organization?   - Would you like to comment on this statement?   14. Because of their key role, HR managers are sometimes assigned supervisors with additional tasks and responsibilities regarding the internal employer brand. Did you have the same feeling for your organization?   - Would you like to comment on this statement?   15. During the pandemic, HR managers recognized that a different leadership style was needed regarding the transfer of the internal employer brand. Some HR managers mentioned that the leadership style that supervisors needed to execute was  more focused on coaching and supporting (e.g. supportive leadership) regarding the internal employer branding.  Did you have the same feeling for your organization?   - Would you like to comment on this statement?   16. HR managers mentioned organizing training sessions during the pandemic to support supervisors in delivering the employer brand to their employees. Did you have the same feeling for your organization?   - Would you like to comment on this statement?   17. Based on the interviews, we could conclude that HR managers mentioned difficulties in transferring the internal employer brand to new employees. How did you familiarize new employees with the internal employer brand during the COVID-19  pandemic? And what aspects of employer branding did new employees struggle with during the COVID-19 pandemic? (open ended questions)  18. Did you reflect on your internal employer branding policy and what were the specific aspects that you reflected on during the COVID-19 pandemic? (open ended question)  19. Based on our interviews, HR managers noticed difficulties in differentiating employer brand communication from other types of communication (e.g. operational communication, COVID-19 regulations, etc.). During the COVId-19 pandemic, did you struggle with this issue and how did you differentiate internal employer brand communication from other communication? (open ended question)  20. During the COVID-19 pandemic, did you assign additional responsibilities regarding the internal employer brand to the supervisors en how were they supported regarding these additional responsibilities? (open ended question)  21. What would you take away from the COVID-19 pandemic to future crises in terms of internal employer branding? (open ended question) |
| Employee check questions (sample 3) | 1. What do you consider to be the core values and objectives of the organization? 2. How would you describe the reputation of your organization as an employer? 3. What does your organization stand for as an employer? 4. What aspects make your organization attractive to current employees? According to you, what are the strengths of your organization as an employer?    - Can you share specific examples of positive experiences you've had working with our company?    - What are the key factors/elements that influenced your decision to work for your company? 5. In the context of the employer branding concept, how did your organization uphold its employer brand during the COVID-19 pandemic?    - Were there specific measures taken to maintain or enhance the employer brand image?    - Were there any shifts or adjustments in the emphasis on employer branding within your organization during this period? If so, in what ways were these shifts or adaptations made? 6. What employer branding actions or initiatives (e.g., employee development, training, team-building activities, flexible work arrangements, etc.) did HR managers undertake during the pandemic to continue supporting employees?    - Can you provide examples of specific ways you felt supported by the organization during the challenging times of the COVID-19 pandemic? This could range from specific policy measures to practical support or other initiatives that eased the impact of the pandemic on your work experience.    - What were the consequences of these initiatives for you? How did this help you navigate through the pandemic? 7. How would you describe the way the company presented itself to you as an employer during the pandemic? (Consider messages, actions, or initiatives used to position the company as an attractive employer. Can you share specific examples that stood out to you during this period?)    - Can you provide specific instances where you experienced the employer branding strategy? Both positive and negative experiences are valuable.    - Are there specific aspects that left an impression on you, both positively and negatively?    - Was there a particular focus from your organization on unity and warmth, and how was this expressed? 8. What impact did the implementation or focus on employer branding (through team-building activities, recognition of achievements, etc.) have on you during the pandemic? (Think about increased engagement, pride in the organization, heightened satisfaction, productivity, etc.) 9. Comparison between HR manager perspective and employee: To what extent did you, as an employee, actually encounter difficulties in connecting with the internal employer brand/values during the COVID-19 pandemic? What were the causes? (Consider factors like remote work, physical distancing, etc.)    - How important do you think recognizing the employer brand is during a crisis, and if so, why? How do you think this translates into employee experiences?    - Were there specific changes or challenges during crisis situations that made recognizing the employer brand more difficult?    - Are there instances where employees, due to difficulties in recognizing the employer brand, might have been more inclined to leave the organization? 10. Comparison between HR manager perspective and employee: To what extent did you, as an employee, actually notice improvements in the content of the internal employer brand due to the COVID-19 pandemic? How did you notice this? 11. Comparison between HR manager perspective and employee: Did you, as an employee, feel that there was more attention given to the internal employer brand during the COVID-19 pandemic, possibly more than before the pandemic? How did you notice this?     - What impact did this have on you? 12. Comparison between HR manager perspective and employee: Did you, as an employee, feel that the internal employer brand was more important than the external employer brand during the pandemic, as some organizations have indicated? Why do you think so/not? 13. Comparison between HR manager perspective and employee: Did you, as an employee, feel that new employees actually had more difficulty understanding and knowing the employer brand during the pandemic? What was the reason for this? 14. Comparison between HR manager perspective and employee: To what extent did you, as an employee, feel that the sense of a common enemy, namely the COVID-19 pandemic, encouraged you to connect with the values of the employer brand? How did this manifest itself? 15. Were there other needs of employees within your organization that were addressed or overlooked by internal employer branding during the crisis period? 16. How was information about the organization as an employer shared with you during the crisis, and how did you experience this?     - Can you share examples of how the internal employer brand within your organization is communicated?     - How would you describe the overall style of this communication during the pandemic? Warm, formal, informal, etc.?     - How often did you receive communication? Through which communication channels was information shared, and was this different from before the pandemic? 17. How did the organization adapt or communicate its internal employer branding during the crisis compared to stable times to you as an employee?     - Were there specific changes noted in how the company communicates about the employer brand? What were these? 18. Comparison between HR manager perspective and employee: Did you, as an employee, actually notice that old methods or strategies to communicate the employer brand among colleagues were not always possible during the COVID-19 pandemic? 19. Comparison between HR manager perspective and employee: To what extent did you, as an employee, experience that communication of the employer brand during the pandemic was more focused on expressing warmth and care? How did you notice this? Can you provide specific examples?     - Can you share specific examples of this? Can you provide examples of moments when communication about the internal employer brand took place in a warm manner? 20. How do you think warm communication about the internal employer brand influenced your personal experience as an employee?     - Are there aspects of this communication that you perceived as positive or negative, and why? 21. Comparison between HR manager perspective and employee: Did you, as an employee, actually experience being overwhelmed with different forms of communication during the pandemic, making it harder for the employer brand communication to stand out among other forms of communication? 22. Comparison between HR manager perspective and employee: To what extent did you, as an employee, observe the introduction of 'target group communication management' during the COVID-19 pandemic, making it easier for you to distinguish and better understand and apply communication of the employer brand in your daily work? 23. Comparison between HR manager perspective and employee: Did you, as an employee, during the pandemic, actually experience that there was less or no communication or feedback from below about the employer brand, and do you think this has led to more misinterpretations of the employer brand?     - Can you share specific examples of moments when misinterpretations about the employer brand occurred? 24. How would you describe leadership within the organization in terms of employer branding during the pandemic? Are there specific characteristics of leaders that stood out? (Extra emphasis on values, living the values, communicating about the values, seeking input from employees, etc.)     - Did you notice leaders actively working to embody the employer brand, and in what way?     - What impact did this have on you as an employee?     - Can you provide examples of moments when leaders contributed to the positive image of the company? 25. How would you describe the leadership style within our organization during the past period? Can you share examples of situations where leaders demonstrated involvement and support?     - Were there specific leadership moments that you think had a positive impact on how the employer brand is experienced, both internally and externally? 26. Comparison between HR manager perspective and employee: Did you, as an employee, actually experience that leaders played a key role in conveying and embodying the employer brand to their employees? 27. Comparison between HR manager perspective and employee: Did you, as an employee, perceive less monitoring of your supervisor? 28. Comparison between HR manager perspective and employee: To what extent did you, as an employee, experience that leaders during the pandemic were given additional tasks and responsibilities related to the internal employer brand, due to their crucial role? 29. Comparison between HR manager perspective and employee: Did you, as an employee, during the pandemic, actually feel the need for a different leadership style regarding the transfer of the internal employer brand, with more emphasis on coaching and support? If so, can you provide concrete examples? 30. Comparison between HR manager perspective and employee: Did you, as an employee, during the pandemic, observe that leadership training sessions followed that support them in conveying the employer brand? 31. Ask if they would like to add anything or have specific suggestions for improving internal employer brand strategies. Is there anything else you would like to add? |
| Drop-off information for 37 HR managers | - Name of the organization: - Name of the contact person - Age of the interviewee: - Gender: - Function: - Degree: - Tenure: - Sector of the organization - Number of locations: - Number of employees |
| Drop-off information for employees | - Name: - Organization: - Age: - Location (if multiple branches): - Seniority/tenure within the organization: - Highest degree: |
| Overview of methods – data sharing analysis | ***Sample 1: interviews HR managers:***  Thematic analysis, combining deductive and inductive elements, was employed using Braun and Clarke's approach for the 37 interviews. Six phases, including data familiarization, coding, theme generation, theme review, defining themes, and implementing themes in the results, were undertaken. The thematic analysis consists of six phases. The initial phase involved data familiarization, including the transcription and (re-)reading of data, as well as the marking of sentences and recording of initial impressions. Subsequently, deductively identified nine themes such as "policy," "policy challenges," and "policy opportunities" were pre-established, initiating the coding process. Initial codes were generated based on emerging patterns in the data, with openness to new insights and contradictions, considering the organizational context. The first author primarily handled coding, engaging in frequent discussions and issue resolution with other authors. After coding 15 interviews, emerging themes like "loss of contact with the employer," "Digitalization," and "New leadership skills" were identified. An analysis was conducted to identify overarching themes at a broader level, leading to the identification of three main themes, six subthemes, and 24 subordinate subthemes. Themes were reviewed, refined, and renamed, with merging for clarity and cohesion. Intercoder reliability was assessed using Krippendorf’s alpha, achieving substantial agreement. The finalization of themes involved merging and consolidation, leading to the implementation of themes (3 main themes, six subthemes, 23 subordinate subthemes) into the results section, accompanied by evidence such as quotes from participants. A member check and employee check were conducted for validation.  ***Sample 2: member check:***  Participants were provided with statements derived from the interviews, and they were asked to express their agreement (yes/no) and provide further elaboration. The results, indicating who affirmed and who negated, have been incorporated into Supporting information: S3_Tab. A commonality was observed among participants, with the majority encountering similar challenges or opportunities.  ***Sample 3: employee check:***  Employee checks were conducted to validate the conclusions drawn from the interviews and ensure alignment with the experiences of the employee group. Interviews were conducted via phone or Microsoft Teams with a total of XX employees. These semi-structured interviews delved into employees' experiences with employer branding strategies during the COVID-19 pandemic, using the 16 statements and results as discussion point. The results of the employee check are presented in the results section and into Supporting information: S3_Tab. |
| Coding scheme | Coding scheme before coding interviews  1. Internal employer branding  1.1 Policy  1.1.1 Challenges  1.1.2 Opportunities  1.2 Internal communication  1.2.1 Challenges  1.2.2 Opportunities  1.3 Leadership  1.3.1 Challenges  1.3.2 Opportunities  Coding scheme after 15 interviews  1. Internal employer branding  1.1 Policy  1.1.1 Challenges  1.1.1.1 Loss of contact with the employer  1.1.1.2 Difficulty in recognizing the employer brand  1.1.2 Opportunities  1.1.2.1 Creation new activities  1.1.2.2 Flexibility regarding the employer brand  1.1.2.3 More focus on internal employer brand  1.2 Internal Communication  1.2.1 Challenges  1.2.1.1 Digitalization  1.2.1.2 Employer brand physic and face-to-face  1.2.2 Opportunities  1.2.2.1 Feedback and Q&A session  1.3 Leadership  1.3.1 Challenges  1.3.1.1 New leadership skills  1.3.2 Opportunities  1.4 Other information  Coding scheme after thematic analysis (before step 4 thematic analysis)  1. Internal employer branding policy  1.1. Challenges  1.1.1 Flexibility and well-being  1.1.2 Onboarding and workplace integration  1.1.3 Remote working and disconnection    1.2 Opportunities  1.2.1 Team building, social connection and alignment with employer branding values  1.2.2 Reflection of internal employer branding and team identity  1.2.3 Retention and strengthening of employer branding values  1.2.4. Flexibility in working hours and environment    2. Internal communication  2.1 Challenges  2.1.1 Digital transformation and challenges  2.1.2 Frequency and form of communication  2.1.3. Loss of informal communication and connectedness    2.2 Opportunities  2.2.1 Message  2.2.2 Digitalization and efficiency  2.2.3 Innovative communication strategies  2.2.4 Flexible and enhanced interaction  2.2.5 Strengthened focus on transparency and executive communication    3. Leadership  3.1 Challenges  3.1.1 Adaptation to remote work  3.1.2 Maintaining team cohesion  3.1.3 Loss of control and monitoring  3.1.4 Extra demand for competencies    3.2 Opportunities  3.2.1 Adaptive leadership training  3.2.2 Fostering communication  3.2.3 Well-being and people centered leadership  3.2.4 Values integration and alignment  3.2.5 Leadership visibility  Coding scheme after intercoder reliability  1. Internal employer branding policy  1.1. Challenges  1.1.1 Loss of connection and remote working  1.1.2 Difficulties with onboarding and new employees    1.2 Opportunities  1.2.1 Reflecting about the internal employer brand  1.2.2 Continuous focus on the internal employer brand  1.2.3 More focus on the internal vs external employer brand  1.2.4 Common enemy feeling    2. Internal communication  2.1 Challenges  2.1.1 Impossible to implement traditional employer brand communication approach  2.1.2 Less bottom-up feedback  2.1.3 Navigating communication overload for internal employer branding    2.2 Opportunities  2.2.1 Emphasis on warmth and care in employer brand communication  2.2.2 Implementation of new digital communication strategies    3. Leadership  3.1 Challenges  3.1.1 Loss of employee monitoring  3.2 Opportunities  3.2.1 Key role in transferring and radiating the employer brand  3.2.2 Assignment of additional tasks  3.2.3 Switch to coaching and supporting  3.2.4 Installments of training sessions |
| Intercoder reliability | To assess intercoder reliability, we closely followed the guidelines of O’Connor and Joffe (2020). The practical coding process began with a deep immersion into the data, involving intensive reading to establish familiarity—a crucial step for understanding the nuances within the information. Subsequently, the corresponding author coded the interviews and, upon completion, developed a comprehensive coding reference framework. The application of this framework was facilitated using NVivo, a qualitative software package.  The initial coder duplicated the coded Excel file, removing code names and providing a "clean" version to the second coder. This enabled the second coder to independently apply codes to the visible data units on the cleaned file using the established coding framework. A subset of codes was randomly presented to another team member, who, with the aid of the coding reference framework, assigned codes to themes (O’Connor & Joffe, 2020).  The subsequent step involved exporting both coded files to SPSS, a statistical software program. The two statistical data files were merged, and SPSS functionalities (kalpha) were utilized to calculate reliability statistics for each code within the coding framework.  Interpreting the results involved referencing a priori thresholds of acceptable reliability, guided by those established guidelines of Landis and Koch (1977). Krippendorff's alpha was also considered due to its flexibility, accommodating multiple coders and various types of data.  Codes that fell below the predefined reliability threshold underwent a thorough evaluation. Ongoing discussions and assessments ensured the continued robustness of our analysis. Potential reasons for interpretational inconsistencies were identified, and necessary adjustments were made to enhance the overall reliability of the coding. This comprehensive approach, from initial immersion to statistical analysis and subsequent refinement, aimed to ensure the consistency and reliability of the coding process. It allowed for a systematic evaluation and adjustment of codes, contributing to the robustness of our research findings. |
