# Supplementary material for: Living the employer brand during a crisis? A qualitative study on internal employer branding in times of the COVID-19 pandemic
Source: PLoS One. 2024 May 13;19(5):e0303361. doi: 10.1371/journal.pone.0303361 (PMC11090342; doi:10.1371/journal.pone.0303361)
Supplement: S3 Table — (DOCX) [file pone.0303361.s003.docx]

S3 Table. Overview and description of the (preset) themes.

| **#** | **Code** | **Description** | **Deductive vs inductive** |
| --- | --- | --- | --- |
| 1 | Internal employer branding policy | Statements about how the COVID-19 pandemic has influenced HR  manager’s perceptions about internal employer branding policy | Deductive |
| 2 | Internal communication | Statements about how the COVID-19 pandemic has influenced the activity: internal communication specifically. | Deductive |
| 3 | Leadership | Statements about how the COVID-19 pandemic has influenced the activity: leadership specifically | Deductive |
| 4 | COVID-19 imposes challenges | Statements related to how COVID-19 imposes challenges regarding internal employer branding policy and its activities | Deductive |
| 5 | COVID-19 creates opportunities | Statements related to how COVID-19 created opportunities regarding internal employer branding and its activities | Deductive |
| 6 | Loss of connection and remote working | Statements addressing challenges, particularly those stemming from the influence of remote work on employer branding identity, underscore the difficulties in team connectivity and spirit. | Inductive |
| 7 | Difficulties with onboarding and new employees | Statements addressing challenges, particularly those arising from the shift to virtual onboarding, underscore the importance of fostering strong bonds and instilling organizational culture. | Inductive |
| 8 | Reflecting about the internal employer brand | Statements addressing opportunities, particularly those stemming from organizations reflecting on and enhancing their internal employer branding, either through redevelopment, acceleration, or refocusing, with a notable emphasis on adapting the delivery of the employer brand in response to remote work challenge | Inductive |
| 9 | Continuous focus on the internal employer brand | Statements addressing opportunities, particularly those emphasizing the pivotal role of retaining and strengthening employer branding values, with a dedicated focus on maintaining continuity in employer branding culture and values and taking proactive measures and activities to reinforce the internal employer brand. | Inductive |
| 10 | More focus on the internal vs. external employer brand | Statements addressing challenges, particularly those underscoring the heightened recognition of the internal aspect of employer branding, with a shift towards fostering strong connections with employees and a greater emphasis on internal brand focus over external recruiting strategies. | Inductive |
| 11 | Common enemy feeling | Statements addressing opportunities, particularly those acknowledging a shared sense of purpose due to a perceived common enemy, underscored the workforce’s strengthened connection with the employer brand. | Inductive |
| 13 | Impossible to implement traditional employer brand communication approach | Statements addressing challenges, particularly those stemming from the rapid shift to digital communication and absence of face-to-face interaction due to the COVID-19 pandemic, which has significantly impacted the sense of connectedness with the employer brand. | Inductive |
| 14 | Less bottom-up feedback | Statements addressing challenges, particularly those highlighting loss of enriched interaction about the employer brand among employees. | Inductive |
| 15 | Navigating communication overload for internal employer branding | Statements addressing challenges, particularly those that revealed concerns about employees struggling to discern important internal employer branding information amid frequent and varied communication channels. | Inductive |
| 16 | Emphasis on warmth and care in employer brand communication | Statements addressing opportunities, particularly those related to strategic shifts in internal communication messages centered around warmth and care. | Inductive |
| 17 | Implementation of new digital communication strategies | Statements addressing opportunities, especially those showcasing organizations navigating internal communication challenges during the COVID-19 pandemic with creative strategies and sustainable communication efforts, like information filtering, demonstrating adaptability, and video communication of the employer brand. | Inductive |
| 18 | Loss of employee monitoring | Statements addressing challenges, particularly the difficulty for supervisors in monitoring individual team members due to the absence of physical presence. They must devise new methods to measure employer branding values among employees, productivity, accountability, and delegate tasks effectively. | Inductive |
| 19 | Key role in transferring and radiating the employer brand | Statements addressing opportunities, particularly those underscoring the crucial role of supervisors in embodying and disseminating the employer brand beyond conventional communication, emphasize the significance of supervisors as influential role models within the organizational structure | Inductive |
| 20 | Assignment of additional tasks | Statements addressing opportunities, particularly those involving supervisors swiftly adapting to managing remote teams. Supervisors are required to demonstrate heightened adaptability and support as organizations navigate changes and assign additional tasks. | Inductive |
| 21 | Switch to coaching and supporting | Statements addressing opportunities, particularly those emphasizing the central theme of prioritizing as a supervisor employees’ well-being. Initiatives, ranging from weekly check-ins to addressing mental health concerns, underscore a people-centered approach that not only fosters a positive work culture but also effectively showcases the organization's commitment to its employer brand. | Inductive |
| 22 | Installments of training sessions | Statements addressing opportunities, particularly those showcasing organizations seizing the chance to provide specialized training for supervisors. It demonstrates a commitment to continuous development and reinforcing the employer brand's dedication to cultivating dynamic leadership. | Inductive |
