# Supplementary material for: Living the employer brand during a crisis? A qualitative study on internal employer branding in times of the COVID-19 pandemic
Source: PLoS One. 2024 May 13;19(5):e0303361. doi: 10.1371/journal.pone.0303361 (PMC11090342; doi:10.1371/journal.pone.0303361)
Supplement: S4 Table — (DOCX) [file pone.0303361.s004.docx]

**S4 Table. Relevant excerpts from the interviews per theme.**

| **Internal employer branding policy** | **Subthemes** | **Representative transcript quotes** |
| --- | --- | --- |
| 1. Challenges | (a) Loss of remote working and disconnection | - That is what you try to do as an organization, to maintain that connection in times of COVID. I am convinced, but that's personal, that physical contact, being together in the office, is still much better than remotely. You can see when something is going on with someone. Now, if something is going on with someone, they will call me, but there is still a certain restraint, a certain distance. I sometimes worry about people too. If you don't feel well, had an argument at home, and you come into the office, well, just sit down for a moment. I was always the first one in the office. When HR people came to say hello, I could see if something was wrong, and we'd sit down for a coffee and discuss what's going on. We don't do that anymore, so that human care has somewhat disappeared. - As mentioned earlier, it really depends on the type of employee. Those who have recently joined the company and are not yet able to work independently may become frustrated more quickly because they can't easily ask a colleague for help when they're stuck. So, it's unfortunate for them. - Yes, I think the challenges primarily revolve around the mental aspect and the distance factor for us - We experienced a significant impact during the first lockdown, where everyone felt a bit isolated, especially between departments like operations, finance, and customer service. We strongly felt that certain individuals were no longer reachable; I couldn't get in touch with them - When people are in open offices, you can hear them talking or interacting with clients, so you get a sense of their internal employer branding. When they are working from home, you have less visibility into that - A lot is happening with regards to internal employer branding, in my opinion, but the real connection with the organization and the work... Many colleagues truly miss that genuine sense of connection |
|  | (b) Difficulties with onboarding and new employees | - But for example, we hired someone in October, and typically, when someone joins us, there's a welcome package waiting, a guided tour of the company, on-the-job training, introductions to the team, and we organize a lunch. None of that has happened now, so that person came to the office for just one day to meet with the manager, but then the rest of the time, they've been working from home. I regularly call them to ask how things are going. We feel somewhat guilty because they're all alone there. They mentioned that it's quite challenging—coming into an organization without knowing the rope - But I also have other observations. We have many people with low seniority, and it's a bit more challenging for them because through Teams or phone, you don't feel comfortable bothering a colleague or your manager for every small thing. So, I notice that the learning process is a bit slower for these individuals, and there's a risk of creating distance in that context - Of course, we have a lot of people, including myself, who have started in the last few months, and if you don't really belong to a team, it becomes quite challenging. It makes them feel very isolated. |
| 2. Opportunities | (a) Reflecting about the internal employer brand | - In terms of content, the employer brand has not changed, but it has been implemented and transferred faster and was repeated more often. Because employees work from home, we had to trust employees to radiate the employer brand correctly, because we cannot know how employees implement the employer brand. In addition, COVID-19 created the opportunity to transfer and position the employer brand differently. The circumstances of the pandemic forced us to do this. - Indeed, COVID has brought both advantages and disadvantages; I believe it served as a catalyst for many companies to completely shift their thinking. So, it has certainly led to both positive and negative outcomes. - As I mentioned before, it might sound a bit strange, but we were in the midst of a cultural transformation, and when something like the COVID crisis happens, there are two possibilities: either you are overwhelmed, and things get even worse, or you say no, maybe this is the opportunity to accelerate certain things, to do things differently. We chose the latter, and I think, most people really appreciated that. From our perspective, I would say, and we also see it in the recent culture survey results, that it has had a rather positive effect on internal branding. - That is actually what I was just saying regarding the latest figures from the culture survey; they even indicated that this evolution happened faster than we expected. I believe this is partly due to the agility we demonstrated to quickly respond to COVID and truly use our values as a compass for our actions. Yes, this translated into better scores in terms of people engagement, so in that regard, I can only reiterate that it had a positive effect. The evolution is somewhat positive and has accelerated positively during the pandemic. |
|  | (b) Continuous focus on the internal employer brand | - I think we have always been a company that pays attention to that. One of the things where we actually need to make a difference (though we're not quite there yet, but it's already encouraged among the managers) is recognizing that due to teleworking, some people may become somewhat isolated. - I believe we have always been a company that pays attention to these aspects. One area where we need to make a difference (although we are not there yet but it has already been encouraged among the managers) is acknowledging that some people, due to telecommuting, feel somewhat isolated. - In terms of content, the employer brand has not changed, but its implementation has been expedited, and it has been reiterated more frequently. With employees working from home, we had to trust them to accurately represent the employer brand, as we cannot oversee how they implement it. Additionally, the circumstances of the COVID-19 pandemic provided an opportunity to convey and position the employer brand differently, and we were compelled to do so. - Integrating new people into a team digitally is not easy, and we are currently putting extra effort into addressing this. We have also prioritized connectivity, launching the buddy project – a mentorship initiative to emphasize this aspect. - One of the most crucial aspects we are currently working on is preserving our identity, branding, etc... We are a team, a society, and we have an identity. We are working on this to avoid losing it completely. - In the context of a COVID-19 case, we spare no cost to minimize the risk for our employees. We have set up a specific COVID-19 consultation body to continuously monitor the actions taken. - Yes, we are trying to consider this in the context of internal employer branding. - We have focused on internal employer branding, and furthermore, it's primarily about ensuring that we stay informed about each other. - We were already engaged with our mission and vision, so those actions have simply continued. I consider them equally important, and I don't think COVID has changed that. For example, in terms of content, it has not changed. |
|  | (c) More focus on the internal vs external employer brand | - During the pandemic, we were more focused on our internal employer brand and ourselves. Before COVID-19, we were focusing on: “How are we perceived in the labor market?” But now, we pay attention to: “How do our employees look at us? How are our employees doing?” So, we are more focused on creating a connection with our employees. - We need to focus more on retention instead of recruiting through external employer branding. We have a good external employer branding strategy, but we need to work on internal employer branding, which became clear during the pandemic. - During the pandemic, we shifted our focus more towards our internal employer brand and our employees. Before COVID-19, our emphasis was on: 'How are we perceived in the labor market?' Now, we pay attention to: 'How do our employees view us? How are our employees doing?' So, we are more focused on creating a connection with our employees." - We need to concentrate more on retention rather than external recruitment through employer branding. While we have a strong external employer branding strategy, it became evident during the pandemic that we need to work on internal employer branding. - Yes, you might say that, for example, the values of the organization/employer, but do you live up to them? Because initially, I didn't believe that; I was skeptical. That was the biggest challenge. Perhaps, Corona has helped to demonstrate that we genuinely want to evolve. To give you a concrete example: Six years ago, the company held fairly conservative views. This evolved; the company became more flexible, from okay: you need to provide some flexibility, and then comes Corona, and you grant flexibility massively. Go ahead, work from home, and if you want to start at 7 a.m. at home, that's fine with me. And if you finish at 4 p.m., I won't see you anyway. So, it all accelerated. It helped us to show that we want to evolve, are evolving, and have evolved into what we have stated. - It can't always happen, but when we organize actions for our staff, we will always say: we are a caring organization for that reason. The year-end gift is to be understood in that context because you have shown a great deal of dedication, and we also want to extend warmth to you, just as you show warmth to our patients. So, we always try to link it to a framework, in terms of values. |
|  | (d) Common enemy feeling | - Well, I think it has strengthened that, because suddenly there was a kind of common enemy, so to speak, and that somehow makes you feel connected, against the common enemy - I believe there was certainly uncertainty, but we were able to quickly turn that, for most people, into a sense of 'Let's go through this together,' creating a strong sense of unity. As an HR professional, I found that really heartening to see. It was like, 'We actually have wonderful people on board—many positive individuals.' They influenced and uplifted those who might have felt left behind. Yes, and that was true for everyone. |
| **Internal Communication** | **Subthemes** | **Representative transcript quotes** |
| 1. Challenges | (a) Impossible to implement traditional employer brand communication approach | - During the pandemic, our employer brand strategy was limited to the most necessary subjects. There was zero interaction and interpersonal contact, which made it difficult to deliver the employer brand to employees - In several departments, there has been some additional investment in organizing team-building activities. I mean, everything is approached from the perspective of not just following the traditional way of monitoring work and measuring results but also addressing the relational aspect. Transmitting the employer brand physically has completely disappeared nowadays. For me, two days a week have opened up where I used to have face-to-face interactions with the employees. Moreover, conveying the values and work practices digitally is quite challenging, especially ensuring that it resonates effectively with individuals. That is why we have dedicated a significant amount of time to fine-tune those conversations digitally. Even though they were conducted over the phone or virtually, we invested a lot of effort to give it extra attention - We had to find other ways to communicate about the values and employer brand with people since they were no longer in the office - During one of those information sessions, quite early on, we warned that you need to be careful, just like with emails. Sometimes, interpretations may not be accurate. - You see fewer signals to begin with because people are at home, and when we meet on Skype or Teams, it's about projects, and they don't really show how they feel. Besides, all those informal channels have disappeared, and doing the same thing in this way is not the same. - Communication was always a challenge. This has become even more difficult due to the COVID-19 pandemic. Nowadays, a message is mainly communicated digitally. Therefore, it is difficult for us to know if the employer brand message is understood and interpreted correctly. - For example, explaining the group insurance, I would have much preferred to do that in a group, where it's much easier to receive certain reactions from colleagues. This won't happen now, and then it's more challenging to maintain that engagement. - Especially in HR, we are people who often sense things. If you see that something is wrong with a person, you call them in, and I don't see that happening today. When your manager or HR representative calls you and asks if everything is okay, it creates engagement. I am seen, I am heard, I am a human being. That is gone, so it will certainly have an impact. I cannot measure it, but it will definitely have an impact. - We used to call people together much more quickly to sit down and discuss something; that doesn't happen now. If there is communication now, it's in very small groups with masks on, and it's a different way of communicating. A lot is being postponed because of that, until the moment when hopefully we can all sit around the table together. But it certainly has an effect on direct contacts. - Very difficult. The loss of face-to-face communication.. |
|  | (c) Less bottom-up feedback | - I, for example, have a team under me, and I have seen those people physically twice. And you can set up virtual meetings, but that is very transactional. It is planned. You don't have those 'off the record' conversations at the coffee machine anymore. You get to know those people a little less, you have less of a connection with them. Certain things also escape you because you're not at the office. So that is very difficult. |
|  | (b) Navigating communication overload for internal employer branding | - Because you can't stand together anymore, you have to say the same thing ten times if you want to reach your people. You have the headset, you have to maintain that distance. There's also a bit of social control, so it has changed. I think it is more individualized. - This is essentially internal communication. Well, we communicate a lot. And that ranges from a very high level of what is important for DEME, what are our targets, what do we stand for, what are we aiming for, to very periodic communication in every possible way. - But indeed, internal communication, I find, has also changed in the sense that it has become faster and more frequent. Because in the past, a lot of information was collected until a certain point when it was communicated. Whereas now, we receive daily updates on certain things. - I must say, we may have made some mistakes at the beginning by wanting to send everything to everyone. Ultimately, people are bombarded with that information. You have to filter that for them |
| 2. Opportunities | (a) Emphasis on warmth and care in employer brand communication | - The channels remain the same, but the language has changed. There has been much more communication around well-being - I found it a very powerful message when your CEO says, “At five o’clock, you can close your laptop.” So, people continued to give, and that is also the pitfall of such an organization where everyone goes for their job at 120%. People can lose themselves. And as an organization, we have a very important role in protecting them. So now the message is: dear people, take your Christmas vacation. Disconnect, take a break, because next year will also be busy, and we do not yet know what awaits us - And then you can refer to that in all-employee sessions, and in communication, there was often reference to those values. 'Care' was one of them, and even when it comes to communicating about a dismissal situation or something similar, we refer to it, based on that care for people. That even in difficult circumstances, we still have an eye for that." - More solidarity indeed, especially in the beginning. I have the impression that it still exists now. There have been a lot of initiatives and communication like: Take care of yourself and your family, and: if there's anything, make sure to let us know and let us know how we can help you. |
|  | (b) Implementation of new digital communication strategies | - The distance or the absence of the employees in the office was a real challenge. We tried to respond to this, so we invested in tools (e.g. info sessions, fun online activities, etc.), tips and tricks, to be able to maintain the delivery of the internal employer brand - We actually switched to video messages at that time, which a colleague and I recorded every Monday. It was a fifteen to thirty-minute session where we provided an update on what was happening in the organization and decisions made regarding instances about the employment experience, and, especially in the beginning, it was very relevant - Because we do have some advantages. We have a total of three sites in Belgium, one site in the Netherlands, and a group structure. If everyone indeed has to keep moving, it's a considerable loss of time. Working in this way has gained a lot of efficiency. However, we all prefer face-to-face contact. We work in HR, so yes, we miss the social aspect tremendously today. But if you want to work efficiently, digital systems have a lot of significance and have taught us a great deal. - So ultimately, in terms of digitization, this is a step forward. COVID has also contributed to that in a positive sense, I think. - In terms of communication, we worked very strongly on: we have an SMS system, so we not only informed people online about the new measures but also through SMS, with a link. It was a platform. So, all employees immediately received an SMS with the new measures that were in effect for us. And that was greatly appreciated. - Now, how do we try to bring that together? One way is to keep the communication line very short so that you know very well what is actually happening on the floor. I think that's important, but also interacting with people, making sure you bring people together at regular intervals. - Things we can do better, how do you work with a helpline that is set up for people who are struggling? - Yesterday, I not only sent the eight best practices to the people. Hey, look, these are eight great tips. Because since COVID, we have been working from home, and it doesn't seem like that's going to change very quickly. - Based on almost everything. What do we have? We purchased headsets. At the moment, I think we have about 15 of them. For example, informational moments on the work floor that used to take place in a meeting room are now held in a quiet space on the work floor. Part of the warehouse where people stand at a safe distance from each other. You can interactively communicate. So, we have invested in tools, tips, and tricks to still be able to maintain that. - We're trying to do a bit of target group management. Communication is often a cascade from top to bottom or from bottom to top, it should work perfectly in both directions. But you also have to avoid that people do not. - Now suddenly, COVID, remote work, so what we have improved since then is that we do a few things. For example, we send out an employee survey every two months where we already ask for input from everyone, and then we ask questions like how are you doing, are there aspects that can be improved, do you have challenges, always around certain aspects that we ask for their feedback on it. - We also have free talks, we, as CEOs, spend a specific time in a room, and people can ask us any question, for instance regarding the work practices, etc., to which we provide answers - We are going to set up something new, an idea that is emerging, is a breakfast with the CEO and the owner. We will invite all newcomers who have started in the past two months. They will then attend a breakfast session from 8-10 persons, where they can ask questions to the CEO or the owner. The aim is to continually create that bond, emphasizing that hierarchical lines should remain very limited, very small. |
| **Leadership** | **Subthemes** | **Representative transcript quotes** |
| 1. Challenges | (a) Loss of employee monitoring | - The extent to which you can control that is much less because people are working from home. - I strongly believe in a climate of trust, that you should treat people from a place of trust and not from a place of mistrust. COVID-19 has resulted in all of us being in lockdown, having to work 100% remotely, and for the controlling types of managers, they have had to let go a bit. They don't know what everyone is doing constantly. Now, for us, this was already the case before because people were not always in the office, but now even more so. In that way, there has been a push for managers to trust people rather than mistrust them, and I think that's the main thing. - I do notice that, from my managerial role, it is very important to do that follow-up every day, to be close to the situation. When people are working next to you, you hear things, you pick up a lot of things, and you can do something about it, but now it's more challenging. |
| 2. Opportunities | (a) Key role in transferring and radiating the employer brand | - We try to keep our communication with people as short as possible. This means there is direct communication from the supervisors to the people on the floor. - Before COVID, we did that with the CMCs. But we also, yes we... The team leaders, so we work in teams, and each team has its supervisor. And those people meet every week with Els, who is our COO. That still continues, even during COVID, but digitally, of course. And in this way, the communication goes to the team members through their team leader. So, we try to maintain as much of normal life as possible here at the office. - We have very good team leaders, who hear from all colleagues every day, even our sales director calls all salespeople weekly. At least half an hour. Just to know how things are, what are the obstacles, how can we help, adjust, so I think that keeping a finger on the pulse is very important again. - We mainly enlisted our team leaders for that, with the message to try to hear from your people regularly, even if it's just to ask how they are doing. - We instructed supervisors to talk and ask (new) employees about the employer brand. And that does establish and fosters that connection with the employer brand. I'm a manager myself and it certainly hasn't gotten any easier in the pandemic. It requires more organization, more structure, more scheduling, and dedicating extra attention to do this - Additionally, our leaders and every responsible person should be the epitome, - We then specified the expected behavior linked to various functions because you can say 'to the point communication,' but what does that mean for someone working in support, what does that mean for a developer... so we wrote down those different values and each time stated what behavior we expected from them under those values, - How did we do that? By demonstrating that we are proactive, that we are fast, you have to live those values. You can put them on a poster and on your website, but you have to live by them. As a management team, we have often conveyed that to our managers, who then pass it on to their people. Transmitting values is a challenge; it's about how we ourselves communicate those values to our people. |
|  | (b) Assignment of additional tasks | - Some supervisors had a more controlling approach when supervising and supporting employees on radiating the employer brand. During the pandemic, supervisors were forced to adapt their behavior – as employees were working from home and direct supervision was not possible. This was a challenge for supervisors – as they were forced to develop people skills and transform their leadership style. - Some supervisors had a more controlling approach when supervising and supporting employees in radiating the employer brand. During the pandemic, supervisors were forced to adapt their behavior – as employees were working from home and direct supervision was not possible. This was a challenge for supervisors – as they were forced to develop people skills and transform their leadership style. - For many people, especially the older audience, if I may say so, some people just said: I no longer do one-on-ones with my employees because I don't want to. That first wave was a significant change. - I think for them, yes, it has become more difficult; it requires different competencies. Leading from a distance requires much more proactivity. Everyone is on their own island at home, and where we used to sit on one large work floor and where you could immediately go to your boss with something or a question, now you have to pick up your phone and wonder if it's worth calling him, schedule a Zoom meeting, so you have much less control as a leader over your people, you have to take much more initiative yourself, and not every leader is blessed with that. - The other aspect is, are you actually, and that was already the case before, going to pick up signals somewhere? Are there people somewhere who are dissatisfied with the organization, is there disappointment? You used to handle that personally by having informal conversations. - In addition, our leaders and every responsible person must be the epitome, but there are also training sessions on how to carry these values forward. Not always easy, but that's how we work. - Actually not. We have our KBI's (Key Behavior Indications) that are outlined. And those are our expectations for each leader. And those values are defined throughout. So actually, in COVID times or in any crisis situation, they should remain the same, but of course, each leader has areas for improvement and focus points, just like every person. But then we, from HR, of course, continue to monitor that, together with the head responsible person at the plant. - So, it becomes difficult to maintain that connection. As HR and as a business leader, we also have to watch over our leaders in the sense of, are they contacting people enough. - Then it is mainly the team leads who are busy making sure they have heard everyone on their team at least once and know what is going on. |
|  | (c) Switch to coaching and supporting | - So, we have invested a lot of time in the past months to have individual conversations with everyone, followed by feedback on those results, and mainly focused on, well, where are you struggling. It was more of the coaching aspect, and it was ideal, or well, not ideal to do it now, but I noticed that some people were having difficulties with certain corona measures, so we were able to discuss that well. - And when you notice that the atmosphere in a team is good, it's not because the manager is such a terrific salesperson or developer. No, usually it's because those are people who pay attention to others, who give that trust. Yes, those are the things that make the difference, and I think you notice that very strongly today. - Uh, that comes up in training. That is a form of people management, and when we talk about a leader, you expect that this person can manage a team and that the focus is on what makes a team stronger. One of the things that comes up in those training sessions is the people management aspect, the link with the company bible. To pass it on to the rest of the team. |
|  | (d) Installments of training sessions | - During this pandemic, we want supervisors to be the example of our employer brand. Therefore, we installed training in the context of: ‘How do you carry those employer brand values during these difficult times? This was not easy to install during the pandemic - Um, through our training called 'Leaders at Work.' Every manager undergoes that training to have the basic principles, and there is a refresh of that training every so often, which includes the values completely. - But we haven't just done that; we also have a training for managers, which is now mainly focused on: how do I delegate to my people remotely? So what is another way to lead your team, to motivate your team, and also how to stay in touch with your people online, through the web, through the new tool. So a kind of awareness of: okay, how can we help our management. |
