# Supplementary material for: Living the employer brand during a crisis? A qualitative study on internal employer branding in times of the COVID-19 pandemic
Source: PLoS One. 2024 May 13;19(5):e0303361. doi: 10.1371/journal.pone.0303361 (PMC11090342; doi:10.1371/journal.pone.0303361)
Supplement: S5 Table — (DOCX) [file pone.0303361.s005.docx]

**S5 Table. Overview of research findings, member check, and employee check.**

|  | **Interviews** | | **Member check** | | **Employee check** | |
| --- | --- | --- | --- | --- | --- | --- |
|  | **Challenges** | **Opportunities** | **Agree** | **Do not agree** | **Agree** | **Do not agree** |
| **Internal employer branding** | HR managers had the feeling that employees had more difficulties connecting with the internal employer brand during the COVID-19 pandemic. (15)^a^ |  | 5 | 1 | 5 | 1 |
|  |  | Due to the COVID-19 pandemic, HR managers were encouraged to reflect on or even improve the content of their internal employer brand. (8) | 2 | 4 | 0 | 6 |
|  |  | HR managers reported that they continued to focus on internal employer branding, sometimes even more than before the pandemic. (14) | 3 | 3 | 6 | 0 |
|  |  | For some organizations, internal employer branding was more important during the pandemic than external employer branding. (3) | 2 | 4 | 3 | 3 |
|  | HR managers had the feeling that it was more difficult to transfer the employer brand among new employees during the COVID-19 pandemic as they have less knowledge and experience with the internal employer brand. (3) |  | 5 | 1 | 6 | 0 |
|  |  | According to HR managers, the feeling of a common enemy (COVID-19 pandemic) encouraged employees to connect with the employer brand. (2) | 2 | 4 | 4 | 2 |
| **Internal communication** | During the COVID-19 pandemic, the traditional methods or strategies to communicate the employer brand among current employees were not always possible. (31) |  | 6 | 0 | 5 | 1 |
|  |  | HR managers reported that because of the pandemic the employer brand communication was more centered on expressing warmth and care. (11) | 5 | 1 | 6 | 0 |
|  | HR managers noticed that during the pandemic they received less or no bottom-up communication or feedback about the employer brand. This may have led to more misinterpretations of the employer brand. (13) |  | 4 | 2 | 4 | 2 |
|  | HR managers had the feeling that employees were overflowed with a lot of different forms of communication during the pandemic. As a result, it was more difficult for employer branding communication to stand out from other communication. (3) |  | 4 | 2 | 2 | 4 |
|  |  | Some HR managers reported they installed “target group communication management” during the COVID-19 pandemic, which facilitated employees to differentiate the employer brand communication and better understand and implement it in their daily jobs. (2) | 1 | 5 | 0 | 6 |
| **Leadership** |  | HR managers noticed that during the pandemic supervisors played a key role in transferring and radiating the employer brand to their employees. (17) | 4 | 2 | 2 | 4 |
|  | HR managers reported that supervisors experienced difficulties when monitoring employees.^b^ (12) |  |  |  | 0 | 6 |
|  |  | Because of their key role during the pandemic, some HR managers assigned supervisors with additional tasks and responsibilities regarding the internal employer brand. (16) | 6 | 0 | 2 | 4 |
|  |  | During the pandemic, HR managers recognized that a different leadership style was needed regarding the transfer of the internal employer brand, focusing more on coaching and supporting. (12) | 6 | 0 | 0 | 6 |
|  |  | Some HR managers organized training sessions during the pandemic to support supervisors in delivering the employer brand to their employees. (13) | 1 | 5 | 4 | 2 |

**^a^**The numbers between brackets are the number of respondents that mentioned this in the interview.

^b^This statement has been added based on new analyses conducted in response to the comments provided by the reviewers. Due to the timing, it was not feasible to incorporate this into the member check process.
