# Supplementary material for: Living the employer brand during a crisis? A qualitative study on internal employer branding in times of the COVID-19 pandemic
Source: PLoS One. 2024 May 13;19(5):e0303361. doi: 10.1371/journal.pone.0303361 (PMC11090342; doi:10.1371/journal.pone.0303361)
Supplement: Attachment — Submitted filename: Response to Reviewers.docx [file pone.0303361.s006.docx]

Dear Editor,

Dear Dr. Georgantzis,

First, thank you for the opportunity to revise our manuscript “*Living the Employer Brand During a Crisis? A Qualitative Study on Internal Employer Branding in Times of the COVID-19 Pandemic”*. We appreciated the many helpful comments and suggestions from you and the reviewers, which have enabled us to further improve the paper.

As you will see in our reply to the reviewers’ comments and our revised paper, we carefully considered and answered all the comments. We believe this submission is a substantial improvement from our initial submission and, thus, we are thankful for your and the reviewers’ comments and the opportunity to address them. A detailed account of all the changes made in reply to the comments is provided in the following pages. In the revised manuscript, titled ‘Revised Manuscript with Track Changes’, changes are highlighted by using red-colored text.

We would like to submit this revised version of our manuscript for publication in *PLOS ONE*.

We look forward to hearing from you.

Sincerely yours,

The authors

**EDITOR COMMENTS**

1. **Thank you for submitting your manuscript to PLOS ONE. After careful consideration, we feel that it has merit but does not fully meet PLOS ONE’s publication criteria as it currently stands. Therefore, we invite you to submit a revised version of the manuscript that addresses the points raised during the review process.**

Firstly, we would like to express our gratitude for your honesty and transparency in writing this review. Additionally, we want to thank you for providing us with the opportunity to revise our paper. After thoroughly reviewing the feedback, we decided to resubmit the paper. Your raised points played a crucial role in significantly improving our work.

2. **When submitting your revision, we need you to address these additional requirements. Please ensure that your manuscript meets PLOS ONE's style requirements, including those for file naming. The PLOS ONE style templates can be found at** [**https://journals.plos.org/plosone/s/file?id=wjVg/PLOSOne_formatting_sample_main_body.pdf**](https://journals.plos.org/plosone/s/file?id=wjVg/PLOSOne_formatting_sample_main_body.pdf) **and**

[**https://journals.plos.org/plosone/s/file?id=ba62/PLOSOne_formatting_sample_title_authors_affiliations.pdf**](https://journals.plos.org/plosone/s/file?id=ba62/PLOSOne_formatting_sample_title_authors_affiliations.pdf)

Thank you for providing the additional requirements and the links to the PLOS ONE style templates. I thoroughly reviewed the guidelines and ensured that my manuscript and title page meets the specified style requirements, including proper file naming. I appreciated your guidance, and I have made the necessary revisions accordingly.

3. **Note from Emily Chenette, Editor in Chief of PLOS ONE, and Iain Hrynaszkiewicz, Director of Open Research Solutions at PLOS: Did you know that depositing data in a repository is associated with up to a 25% citation advantage (**[**https://doi.org/10.1371/journal.pone.0230416**](https://doi.org/10.1371/journal.pone.0230416) **)? If you’ve not already done so, consider depositing your raw data in a repository to ensure your work is read, appreciated and cited by the largest possible audience. You’ll also earn an Accessible Data icon on your published paper if you deposit your data in any participating repository (**[**https://plos.org/open-science/open-data/#accessible-data**](https://plos.org/open-science/open-data/#accessible-data)**).**

Thank you for sharing this valuable information. I highly appreciate the importance of open data practices. However, I'm currently unable to deposit my raw data in a repository as my study participants have not given consent for such sharing. I want to respect their privacy and confidentiality. However, as I do recognize the value of open research practice, I have chosen to deposit a minimal data set in the supplementary materials (see Supporting information ‘S3_Tab’) and displayed some relevant excerpts from the interviews (see Supporting information ‘S5_Tab’). This includes relevant excerpts of the interview transcripts organized by codes.

4. **You indicated that ethical approval was not necessary for your study. We understand that the framework for ethical oversight requirements for studies of this type may differ depending on the setting and we would appreciate some further clarification regarding your research. Could you please provide further details on why your study is exempt from the need for approval and confirmation from your institutional review board or research ethics committee (e.g., in the form of a letter or email correspondence) that ethics review was not necessary for this study? Please include a copy of the correspondence as an ""Other"" file.**

Thank you for your insightful comment and your request for additional information regarding the ethical approval of my study. I understand the importance of ethical considerations in research and appreciate the attention given to this matter. Allow me to provide further clarification on why ethical approval was deemed unnecessary for my study.

First of all, it is crucial to highlight that my research is conducted with a rigorous commitment to ethical principles. I have thoroughly examined the ethical code of my faculty, ensuring that my study adheres to its guidelines. The study is meticulously shaped with a deep understanding of the ethical guidelines established by the faculty. Specifically, the procedures for securing voluntary informed consent from participants, ensuring anonymity during data collection, and allowing participants to withdraw without consequences are intentional reflections of the explicit directives outlined in the faculty's ethical code.

Furthermore, I have included a segment from my correspondence with the institutional review board, confirming their agreement that formal ethical approval was unnecessary for the study. The attached "Other" file contains the official documentation supporting this exemption. The rationale for this decision stems from the qualitative nature of my research, which involved interviews with HR managers regarding employer branding during the pandemic. Given the characteristics of the study, I concluded that it falls into the category of low-risk research. The research design adopts a non-invasive, non-sensitive approach, avoiding loaded questions to mitigate potential harm or discomfort to participants. Participation in the study is entirely voluntary, with participants receiving detailed information about the study and providing informed consent. They understand that they can withdraw at any point without facing negative consequences. Additionally, the data collection process ensures participant anonymity, with all collected data anonymized and securely handled to protect confidentiality. This comprehensive ethical approach aligns with the principles of safeguarding participants' rights and well-being in research, contributing to the overall integrity of the study.

I trust this additional information addresses your concerns. If you have any further questions or require additional documentation or correspondences, I would be happy to provide them.

5. **In your Data Availability statement, you have not specified where the minimal data set underlying the results described in your manuscript can be found. PLOS defines a study's minimal data set as the underlying data used to reach the conclusions drawn in the manuscript and any additional data required to replicate the reported study findings in their entirety. All PLOS journals require that the minimal data set be made fully available. For more information about our data policy, please see** [**http://journals.plos.org/plosone/s/data-availability**](http://journals.plos.org/plosone/s/data-availability)**. Upon re-submitting your revised manuscript, please upload your study’s minimal underlying data set as either Supporting Information files or to a stable, public repository and include the relevant URLs, DOIs, or accession numbers within your revised cover letter. For a list of acceptable repositories, please see** [**http://journals.plos.org/plosone/s/data-availability#loc-recommended-repositories**](http://journals.plos.org/plosone/s/data-availability#loc-recommended-repositories) **. Any potentially identifying patient information must be fully anonymized.**

**Important: If there are ethical or legal restrictions to sharing your data publicly, please explain these restrictions in detail. Please see our guidelines for more information on what we consider unacceptable restrictions to publicly sharing data:** [**http://journals.plos.org/plosone/s/data-availability#loc-unacceptable-data-access-restrictions**](http://journals.plos.org/plosone/s/data-availability#loc-unacceptable-data-access-restrictions) **. Note that it is not acceptable for the authors to be the sole named individuals responsible for ensuring data access. We will update your Data Availability statement to reflect the information you provide in your cover letter.**

Thank you for bringing this to my attention and we appreciated the clarification regarding the required data set for PLOS ONE. To address this matter, we have included a 'minimal data set' in supporting information (see ‘S3_Tab’, ‘S4_Tab’, and ‘S5_Tab’). The underlying results described in my manuscript are appropriately shared in compliance with PLOS ONE guidelines. We have included the following in the supporting information files:

- Data Availability Statement: We have included a data availability statement specifying how and where people can find the data. We also explained clearly whether specific transcripts or data portions cannot be shared due to ethical considerations or participant consent restrictions.
- Description of Analysis Method: We have shared specific methods used to analyze the qualitative data, including the coding scheme, coding methods, thematic analysis, or other pertinent procedures.
- All Qualitative Codes: We have provided all the applied codes along with a description of how these codes were applied to the data.
- Description of Data Analysis Process: We have given an overview of the qualitative data analysis process, outlining steps and any iterative or cyclical processes.
- Excerpts of Transcripts: We have incorporated relevant excerpts from the qualitative interviews that played a crucial role in shaping the conclusions presented in the article. These excerpts are systematically organized according to relevant codes (see ‘S5_Tab’).
- Member check and employee check results: Results of the member check and employee check are presented in the Supporting Information ('S4_Tab'). The raw data from the member check can be obtained by requesting it from the authors. The raw data from the employee check consists of interview transcripts and cannot be publicly shared, as employees have not explicitly consented to such sharing. However, specific data points showing which employees agreed or disagreed can be shared upon request by contacting the authors. If necessary, we can include these materials in supporting information.

6. **Please ensure that you include a title page within your main document. You should list all authors and all affiliations as per our author instructions and clearly indicate the corresponding author.**

Thank you for your valuable feedback. We appreciated your attention to detail. In response to your comment, we have included a title page within the main document as per your guidelines.

7. **Please include your full ethics statement in the ‘Methods’ section of your manuscript file. In your statement, please include the full name of the IRB or ethics committee who approved or waived your study, as well as whether or not you obtained informed written or verbal consent. If consent was waived for your study, please include this information in your statement as well.**

Thank you. In response to your comment, we have incorporated our complete ethics statement into the 'Methods' section of the manuscript file in the additional section, titled ‘Ethical approval and consent’. This statement furnishes details on the Institutional Review Board that approved our study, providing its full name. We have also specified that informed written and verbal consent was obtained. Here is the relevant excerpt:

*“Our study adhered to the stringent ethical protocol of the authors’ university. Therefore, obtaining specific ethical approval was not required, which was confirmed by the university’s Committee Ethical Affairs Faculty of Economics and Business Administration (Ghent University). Participants from the interviews, member check, and employee check provided both written and verbal informed consent, involving a detailed explanation of the study's objectives, purposes, and procedures. Participants were informed about key aspects, including the voluntary nature of their involvement, the option to withdraw at any point, the recording process with privacy safeguards, permission for data processing, and the opportunity to access the research findings. Only the first author has access to the personal information of the individual participants. Anonymity and confidentiality measures were rigorously implemented, employing code numbers, a distinct file linking personal information to these code numbers, anonymized data files, restricted access to data, and safeguards to protect any identifiable elements.”* (see page 12-13, lines 295-308)

**REVIEWER 1**

1. **The study topic is interesting and deserves to have a chance as a publication to understand more about employer branding.**

Thank you for recognizing the significance of our study topic on employer branding. We are committed to providing valuable insights and contributing to the understanding of this subject.

2. **However, with the current writing, I feel that theoretical background has not been deeply analyzed. I suggest the authors to improve the section as well as the introduction part. The technical details should be expanded and clarified to ensure that readers understand exactly what the researchers studied.**

Thank you for your thoughtful suggestion. In response to this suggestion, we have deepened the introduction and theoretical background. First, we have specified in the introduction the necessity of research on internal employer branding during a disruptive event and why we opt for internal communication and leadership as important touch-points. We have included and modified the following paragraphs*:*

“*Since the expression ‘war for talent’ suggested by McKinsey, organizations are applying different approaches to compete for talent. Literature has proposed employer branding as an effective organizational strategy to win the ‘war’ by attracting and retaining talented employees [1]. Employer branding aims to differentiate the organization, internally and externally, through its unique employment experience and attractiveness as an employer [2, 3]. To enhance attractiveness, creating and promoting a strong employer within the organization (i.e. internal employer branding) and externally promoting the employer brand through recruitment strategies (i.e. external employer branding) are crucial elements.*

*Research has extensively studied the external promotion of the employer image. Internal employer branding, however, has received little attention compared to external employer branding [4]. Backhaus and Tikoo [5] described internal employer branding as a strategy that creates, promotes, and delivers a distinctive and attractive image as an employer (i.e., employer brand) to employees. To achieve an effective internal employer branding approach, the employer brand has to be present in each aspect of the organization [6]. Hence, employees should experience the internal employer brand through multiple touch-points, such as internal communication and leadership [7, 8]. These aspects, already established as important during stable times, form integral components of an effective internal employer branding strategy. Prior research suggests that a strong internal employer brand relates positively to employees’ attitudes and reduces turnover intentions [9, 10]. However, previous studies typically focused on employee perceptions, largely ignoring the crucial role that HR managers or employer brand managers play in crafting and implementing internal employer branding strategies [11]. Hence, we know little on how strategic decisions with regard to internal employer branding are made and implemented.*

*Furthermore, to extend our knowledge of internal employer branding, scholars like Lievens and Slaughter [12] advocate for exploring the employer brand amid unstable times, emphasizing that current literature predominantly addresses stable environments. This imperative gains significance in an era where crises, fueled by factors such as the rise of social media and accelerated media flows, occur more frequently, amplifying threats to reputations [13, 14]. During a crisis, the employer brand can be vulnerable to damage and negative influences [15]. Crises intensify the pressure on organizations to make strategic decisions. These decisions impacts the immediate well-being of employees that experience enhanced levels of frustration, uncertainty and need for information during a crisis [16]. Additionally, during crises, strategic decisions can influence employees' perceptions of the employer brand, which may prompt questions about the alignment between the organization’s stated employer brand values and the actions taken during challenging circumstances. For instance, if an organization claims to be a warm and supportive employer, will this promise hold on when things get tough and layoffs may seem necessary? Moreover, is the employer value proposition still relevant during a crisis or do certain other aspects become more important, such as creating more togetherness among employees or focusing on the operational functioning? Furthermore, some employer branding practices might become difficult or even impossible in the midst of a crisis, when resources might be lacking or social media unavailable. Navigating this delicate balance between addressing immediate operational needs and preserving the long-term reputation of the employer brand might pose significant challenges for organizations and HR managers are likely to play a crucial role in shaping the internal employer brand during such transformative events.*

*The COVID-19 pandemic, with its unprecedented disruptive nature and its impact on employees’ careers and well-being [17, 18] presents a unique opportunity to delve into the intricacies of internal employer branding during a crisis. This crisis not only necessitated organizational adaptations but also left an indelible impact on employees [19, 20]. During the pandemic, organizations were forced to adopt new working routines and policies to ensure business continuity. At the same time, these restrictions and working routines threatened employees’ well-being due to the inability to separate work-private life, the feeling of job insecurity, and loneliness [17, 18, 21]. It created an environment where employees experienced uncertainties, changing work patterns, and feelings of isolation. Thus, the COVID-19 pandemic, with its challenges, has been a disruption that overturned existing working arrangements [22] and threatened employees’ well-being [23]. Therefore, examining internal employer branding during the COVID-19 pandemic provides a suitable context to understand how HR managers navigate challenges and safeguard their organization’s employer brand in times of crises.*

*This study aims to deepen our understanding of how internal employer branding is influenced by disruptive events, specifically exploring the strategic decisions made and implemented by HR managers during the pandemic. We also aim to investigate the role of the key internal employer branding touchpoints internal communication and leadership during a crisis, as they may be vital for adapting employees to new conditions and challenges [24]. For instance, the provision of emotional support, empathy, a relationship-oriented communication, and supportive leadership might enhance a sense of togetherness, trust, and support among employees, potentially fostering commitment to the employer brand during the pandemic.*

*To this end, in-depth interviews were conducted with 37 HR managers from 37 Belgian organizations that varied in sector and size. To validate our findings, we conducted a member check with six other HR managers from different organizations and an employee check with six employees from the organizations that participated in the study. On a theoretical level, our study makes at least two significant contributions. First, we shed light on how HR or employer brand managers make and implement strategic decisions regarding internal employer branding. We thus extend prior research that has mostly looked at employer brand perceptions held by employees and answer the call for more organization-level, strategic HRM research. Second, our research explores the dynamics of internal employer branding during crises, offering insights into the challenges and opportunities they may create and how HR managers respond to them. These insights complement prior research conducted in stable times. Regarding practical implications, our findings are useful for HR managers wanting to learn from their colleagues and how they have handled internal employer branding during disruptive events. It can make them more aware of the strategic considerations involved and their own decision-making process.”* **(**see pages 2-6, lines 44 – 126)

Furthermore, we also expanded the theoretical background with more literature insight in the touch-points. This elaboration provides a comprehensive understanding of the significance behind investigating internal communication and leadership, especially in the context of potential impact from disruptive events. We modified following paragraphs in the chapter “Internal Employer Branding”:

“*Internal employer branding is the promotion of what makes the organization different and desirable as an employer to an internal audience [25, 26]. Originally, internal employer branding stems from a marketing concept, called internal branding. According to Barros-Arrieta and García-Cali [27], internal branding is “a strategy to promote the brand within the organization to ensure that employees adequately deliver the brand promise to external customers” [36, p. 136]. Moreover, internal branding aims to affect customers’ experiences and perceptions of the brand through employee knowledge, commitment, and behavior [28, 29]. While internal branding promotes a brand, product, or service among employees and intends to motivate them to communicate this to external customers, internal employer branding promotes and radiates an image of what makes the employer unique and desirable among current employees. Therefore, internal employer brands describe how internal audiences perceive the organization's employment offer, unique work experiences, and employer values.*

*Scholars have proposed that the employer brand should emerge in every part of the employment experience [6]. In every operational and interpersonal touch-point with employees, the employer brand should be clearly present and consistently managed [30]. Particularly, internal communication and leadership are important touch-points to transfer the employer brand to employees. Staniec and Kalińska-Kula [31] suggested that these are essential touch-points to facilitate the delivery of the employer brand. However, so far limited empirical research has examined the role of internal communication and leadership in internal employer branding. Moreover, in literature there is no insight yet in how these touch-points might be altered by HR managers during a disruptive event to align with the requirements of the employer branding strategy.” (see pages 7-8, lines 155 – 178)*

We modified following paragraphs concerning the touch-points “Internal communication” and “Leadership”:

*“Internal communication, as defined by Kalla [32], encompasses all formal and informal exchanges of information within an organization, playing a crucial role in daily and strategic activities [33]. Recognized as pivotal, it is fundamental for successful employer brand development and management, ensuring consistent perceptions among internal and external stakeholders [34]. Internal communication about the employer brand, managed by HR managers, serves as a tool for conveying employment advantages, sharing core values, aligning employee behavior with the employer brand, and fostering commitment and loyalty [6, 35, 36, 37].*

*Moreover, it aids in developing committed and loyal employees who identify with the employer brand values [27]. According to Itam et al., [38], internal communication reduces information asymmetry among current employees by sharing the organization’s mission and values. HR managers view internal communication as collaborative employer brand crafting [39], facilitating the exchange of information and unique values related to the employer brand [33]. It plays a pivotal role in shaping employee attitudes toward employer branding [7, 28]. Effective communication is thus a key factor in successful employer brand development and management, ensuring uniform perceptions among internal and external audiences and employees’ attitudes toward the employer brand during stable times.*

*In times of crisis, organizations face the challenge of reassessing traditional approaches that may prove inadequate under the pressures of such situations. The COVID-19 pandemic introduces new working practices, particularly the transition to remote and hybrid work, potentially influencing how employees perceive the employer brand. Recognizing the need for a deeper understanding, organizations may consider adopting a communication strategy focused on fostering relationships, unity, and a sense of togetherness, aiming to ensure employee satisfaction during a crisis. Nelke [40] shows that this can help to increase trust among employees. Moreover, according to Sinčić Ćorić and Špoljarić [33], satisfaction with internal communication is important for employees to foster engagement and employee satisfaction. Therefore, to craft an effective internal communication strategy crucial for shaping, understanding, and fostering a positive perception of the employer brand during a disruptive event, HR managers may need to adapt the delivery approaches, modify content, or alter the manner in which it is presented to employees.”* (see pages 8-9, lines 179 – 209)

“*Supervisors can influence, motivate, and enable employees to achieve a specific goal [41]. Leadership concerning employer branding includes all the efforts of leaders to disseminate the employer brand principles to employees, which entails showing the work culture and unique work practices. HR managers rely on supervisors to convey the employer brand to employees, forming a crucial partnership in delivering the employer brand to employees. Zeesahn et al., [42] emphasized that organizations should focus on both leadership and internal employer branding to retain and attract employees. Moreover, leadership has a significant part in creating an employer brand climate and establishing an inner atmosphere which impacts employees’ attitudes [43].*

*Barrow and Mosley [44] emphasize supervisors’ pivotal role in developing and communicating the employer brand. For instance, supervisors can actively embody the employer brand by sharing personal experiences that reflect the employer brand values during team meetings. Concurrently, supervisors, as suggested by Biswas and Suar [45], can provide clear directives, like implementing regular team-building activities, to showcase distinctive work practices. These visible leadership actions not only influence how employees perceive the internal employer brand, values, and culture, but can also support them in incorporating these aspects into their daily tasks [45]. For instance, during a daily team meeting, employees can proactively contribute to a warm and collaborative atmosphere by sharing positive experiences related to teamwork or recognizing a colleague's effort. This daily practice aligns with the employer brand's emphasis on collaboration and fosters a positive workplace culture. Therefore, to achieve a strong internal employer brand, supervisors have to actively “live the employer brand”, and display the employer brand in their interactions with employees.*

*Nevertheless, during a disruptive event, supervisors can rise to the challenge while other supervisors will struggle to manage the crisis [46]. Supervisors are relying on their instincts and insights, supported by HR managers, to ensure their employees feel supported. Dirani et al., [46] concluded that when employees face challenges in a new work environment, supervisors must become more flexible to adjust their employees to the new altered work environment. Moreover, supervisors may need to implement targeted approaches to address the likely impact of a crisis on employees' motivation, satisfaction, and overall well-being. This could involve strategies such as transparent communication, empathetic leadership, and tailored support mechanisms [47]. As such, to foster employer brand-aligned leadership during turbulent times, it is crucial to explore how HR managers guide supervisors in navigating challenges and promoting the employer brand among employees.” (see pages 9-10, lines 210 – 243)*

3. **In the materials and methods section, I did not see the interview guides. I am particularly curious on how the "questions were continuously adapted..." (lines 234-235).**

Thank you for your valuable feedback. In response to your inquiry regarding the interview guides, we recognize that we did not explicitly incorporate them into the Materials and Methods section. However, we did outline the structure of the interview guides. To accommodate space limitations, we have now included both the original interview questions as the adapted question in the supplementary material file (see Supporting information ‘S3_Tab’). Questions were adjusted based on the insights gained from the interviews. Following the completion of five interviews, we transcribed the recorded sessions. Notably, during these interviews, we observed HR managers emphasizing the significance of supportive leadership regarding employer branding. Subsequently, we incorporated specific questions addressing concrete actions of supportive leadership into our interviews. Thus, our line of questioning was consistently refined and adapted throughout the research process. Following paragraph was modified:

“*The interviews were based on insights retrieved from a literature review. Before we started the interview, we made sure that there was a common understanding of the concept of internal employer branding by asking what they understood regarding this topic and providing a definition of this concept. The interview guide covered three main categories: (a) internal employer branding, (b) internal communication about employer branding, and (c) leadership in function of employer branding (see ‘S3_Tab’). Furthermore, in line with guidelines, the initial interview questions were regularly updated based on the presented information by HR managers during this data-collection process [48]. We applied the principles of data saturation and information redundancy. Our data collection ended when additional interviews did not bring new information. This was achieved after about 30 interviews, but seven control interviews were still conducted to be sure. Participants were instructed to provide honest answers based on their experiences during the COVID-19 pandemic.”* (see page 14-15, lines 345 - 356)

4. **Also, not particularly convinced on how the authors doing the population and sample choices. Although it is briefly mentioned in the lines 217 - 220, but when we talked specifically about COVID 19, a lot of business will experience significantly different with others (e.g., hospital workers were busier while many restaurants closed their business; or business in urban, big cities vs in more rural locations).** **With a relatively big number of respondents, I would expect more thoroughly results.**

Thank you for your insightful comment. Your attention to the population and sample choices in our study is highly valued. We acknowledge the concern regarding organizations like restaurants and hotels, which could potentially impact the interpretability of our results. As outlined in lines 285-293, our deliberate decision to exclude certain organizations, such as restaurants and hotels, was driven by the specific focus on entities that operated during the pandemic.

Given the scarcity of research on internal employer branding, we adopted a thematic analysis approach, emphasizing the necessity to explore a variety of experiences across diverse organizations. This strategic choice aims to comprehensively capture the nuances of this relatively unexplored phenomenon. Drawing on Braun and Clarke [49], we underscore the significance of engaging a diverse participant group, aligning with the nature of thematic analysis that thrives on identifying patterns of meaning across multiple cases. Therefore, we specified in our manuscript how and why we sampled an opted for this population.

*“As the perspective of HR managers about internal employer branding during unstable times lacks clear understanding and knowledge [11, 12], a qualitative approach was adopted. Qualitative research - due to its innate explorative nature - allows for more detailed insights into a topic, as it builds upon the perceived realities of its target population [50]. It enables us to capture the nuances of this relatively unexplored topic. Therefore, this study specifically delved into the perceptions and experiences of HR managers regarding their internal employer branding strategies and practices during the pandemic.*

*Our participant selection involved purposeful and maximum variation sampling [51]. Purposeful sampling was utilized to identify and choose participants whose characteristics align with the focus of our investigation [52]. It is particularly useful when the researcher intends to recruit participants who possess specific information based on their professional experiences [53, 54]. For the current study, we intended to recruit participants that were responsible for (internal) employer branding. Furthermore, since the objective was to enhance our comprehension of the phenomenon [49, 55], we intentionally sought diversity through maximum variation. Consequently, we selected organizations that maximally differed on two conditions that may influence employer branding activities, namely organization size and sector [56, 57].*

*To facilitate open sharing of experiences and perceptions, including potential negative views or personal accounts, we chose individual semi-structured interviews to collect the data [58, 59]. The interviews were carried out by two master students in HRM and a doctoral researcher. We trained students’ interview techniques before and during the interviews. Using our network, we contacted organizations via email in which we specified the topics to be addressed. The interviews were conducted through video calls, as teleworking was the norm at that time, and were recorded*.*”* (see pages 13-14, lines 320 – 344)

Secondly, we appreciate your expectation for more in-depth results, especially given the substantial number of respondents in our study. To meet this expectation while acknowledging page constraints, we have opted to furnish a comprehensive account in the supporting information ‘S3_Tab’. This entails presenting our coding tree, providing a step-by-step breakdown of our thematic analysis conducted in NVivo 8, calculating the intercoder reliability (details specified in comment 6), and incorporating excerpts of transcripts to illustrate the basis for our conclusions (refer to supporting information see ‘S5_Tab’). Furthermore, we modified following paragraphs in the method section to show our rigorous data-analysis process:

“*We implemented the thematic analysis of Braun and Clarke [72] to discover patterns in the data. Our research design has both an inductive and deductive component, therefore, thematic analysis is the most appropriate approach. The interviews were structured around themes identified in prior literature [73]. We were receptive to investigating various subthemes associated with internal employer branding, internal communication, leadership, and the emergence of new themes. See supplementary information in 'S2_Tab' for an overview and description of the (sub)themes and 'S3_Tab' for insights into data analysis.*

*The thematic analysis consists of six phases and was performed using NVivo 8. In the first phase, we familiarized ourselves with our data by transcribing and (re-)reading the data. During this step, we marked sentences and wrote down our first impressions. In the second phase, prior to initiating the coding process, we pre-established nine themes deductively, including categories such as "policy," "policy challenges," and "policy opportunities". Subsequently, initial codes were generated based on patterns that emerged from the collected data. During the initial coding, we were open to new insights and contradictions and took into account the organizational context. The first author was mainly responsible for the coding, but the coding and themes were frequently discussed with the other authors, and issues were resolved in consensus. In the third phase, following the coding of 15 interviews, our initial focus involved identifying new subthemes within our coding. Themes like "loss of contact with the employer," "digitalization", and "new leadership skills" surfaced during this phase. Upon completing the coding for all interviews, we conducted an analysis by shifting our attention to a broader level of themes. This involved identifying overarching themes, such as various types of employer brands, organizational size, and sector. We built a thematic map of the codes and reflected on the relationship between these themes. At the end of this step, we identified three main themes (e.g. “internal employer branding”, “internal communication”, and “leadership”) and six themes (“policy challenges”, “policy opportunities”, “internal communication challenges”, “internal communication opportunities”, “leadership challenges”, and “leadership opportunities). As thematic analyses allow for the emergence of inductive themes, we identified a total of 24 inductive subthemes within the deductive (main)themes. These included topics like "flexibility and well-being," "retention and strengthening of employer brand values," "loss of informal communication and connectedness," "message," and more. In the fourth step, we reviewed, refined, and renamed the themes and looked for overlapping themes. While retaining the three main themes and six themes, we settled on 23 subthemes. Notably, we discussed and opted to merge the themes "leadership visibility" and "fostering communication" into a single theme, named “Leadership visibility through communication”. Next, in the fifth step, we assessed intercoder reliability using the Krippendorf’s alpha (Kalpha) statistic, a commonly employed measure for such evaluations [74]. A random subset of quotes was provided to another team member. Utilizing the coding reference framework, which includes detailed content information for the subthemes (see 'S3_Tab'), the team member assigned codes to the subthemes following the method outlined by O’Connor and Joffe [74]. This process yielded a Kalpha value of .81, signifying substantial agreement. Ongoing discussions and assessments ensured the continued robustness of our analysis. We made the decision to merge 23 subthemes into 16 subthemes, for instance “Reinforcement of internal employer branding” and “Retention and strengthening of employer brand” consolidating them into a subtheme renamed as "Continuous focus on the internal employer brand". In the sixth step, we implemented the subthemes in the results section and elaborated on these subthemes by providing evidence (i.e. quotes from participants) (see ‘S5_Tab’), conducting a member and employee check*.*”* (see pages 16-18, lines 381-428)

Moreover, to reinforce the credibility of our analysis, we performed a member check with six diverse HR managers, verifying results and ensuring consistency. Extending validation, we conducted an additional check with employees, as detailed in the method section. Demographic data for participants are in supporting information ‘S1_Tab’. We interviewed six individuals to gather their perspectives on HR managers' strategies for internal employer branding.

“*Finally, we conducted employee checks to ensure that the conclusions derived from the interviews were experiences that were recognizable to the employee target group [71]. This allowed us to verify whether members of the target group of internal employer branding agreed with our findings. Moreover, this enabled us to supplement our results with their input. As such, in December 2023, we carried out an employee check to authenticate and confirm the research findings from the employees' standpoint. We reached out to all 37 organizations and conducted interviews with six employees, each lasting between 25 and 35 minutes. During these semi-structured interviews, we inquired the participants about their experiences with the strategies and practices related to employer branding that were implemented during the COVID-19 pandemic, using the 16 statements and results as a basis for discussion. Prior to posing these questions, we ensured that employees had a clear understanding of the concept of employer branding. Subsequently, employees were given the opportunity to elaborate on these statements. Consult 'S1_Tab' for participant details.”* (see pages 15-16, lines 367-380)

Furthermore, we briefly incorporated and addressed their insights in the results section and discussion, albeit not the primary focus of our study. However, we recognize the significance of delving into the employee perspective for a more comprehensive understanding. Subsequent paragraphs have been included in the results section.

*“To gain insights from employees, we conducted a check involving individuals from the same organizations as those interviewed. Overall, employees validated the findings pertaining to internal employer branding, internal communication, and leadership (see 'S4_Tab'). Participants highlighted challenges in perceiving the employer brand due to physical distance, resulting in diminished connection and commitment. Traditional methods of communicating the employer brand to current employees encountered pandemic-related obstacles, as indicated by HR managers and corroborated by employees. The employee check additionally observed a shift toward warmth and care in employer brand communication.*

*HR managers noted a decline in bottom-up communication, potentially leading to misinterpretations, a sentiment supported by the employee check. Furthermore, HR managers discussed difficulties in monitoring employees and encouraged supervisors to regularly check in with their teams, a point substantiated by the employee check. Employees reported an increase in their interactions with supervisors.*

*In addressing the need for a different leadership style during the pandemic, HR managers emphasized coaching and support in conveying the internal employer brand. However, many employees did not perceive this as supervisors delivering the employer brand more frequently; instead, they acknowledged increased support through regular check-ins, assistance, and flexibility. This suggests that while positively influencing the employee experience, supervisors' actions may not be explicitly seen as a direct communication or embodiment of the employer brand. Employees were cognizant of training sessions for supervisors.*” (see pages 35-36, lines 836 – 857)

Reanalyzing 37 transcripts in NVivo 8 has provided valuable insights that enhance our findings and discussion. Examining the employer brand context allows us to offer fresh perspectives on internal employer branding strategies during a pandemic. Notably, despite different focuses in the employer brand (warmth vs competence), HR managers encountered similar challenges. However, divergences emerged in their approaches, heavily influenced by their employer value proposition. Subsequent paragraphs, expanding on these observations, have been incorporated into the results section.

“*To incorporate contextual elements, we investigated whether distinct types of employer brands—warm versus competent, encountered different challenges and opportunities. We initiated a contextual analysis by first soliciting descriptions of internal employer branding and the content of employer brands during the interviews. Organizations that articulated a familial, warm, or social emphasis in their descriptions were categorized as warm employer brands. Conversely, those highlighting aspects such as training opportunities, employee growth, and competence development were classified as competent employer brands. Out of the 37 organizations analyzed, we successfully categorized 28, identifying 11 as warm employer brands and 17 as competent employer brands. This categorization formed the foundation for further exploration, allowing us to investigate whether and how these distinct employer brand types encountered unique challenges or opportunities amidst the pandemic.*

***Content***

*Examining the content of these two types of the employer brand, it is noteworthy that challenges across the two types of employer brands appear largely similar. However, the key difference lies in their content. Competence-oriented employer brands prioritize formality, competencies, and efficiency within a professional framework. They focus on efficiency, innovation, and digital transition. In contrast, warmth-oriented brands emphasize personal connections, care, well-being, employee recognition, and a positive atmosphere during the pandemic. They present well-being as part of a broader positive branding strategy, utilizing symbolic language and digital communication. The communication style is informal, fostering an open atmosphere and focusing on individual well-being to enhance team culture and relationships. Despite facing common challenges, each approach customizes its employer brand transfer and communication style according to its unique employer value proposition, reinforcing its distinct employer brand identity.*

***Approach***

*Both competence-oriented and warmth-oriented employer brands showed a strong commitment to internal branding during the pandemic, emphasizing on engagement and employer brand values. Competency-focused organizations strategically addressed the challenges, prioritizing innovation, and preserving organizational culture. In contrast, warmth-focused organizations prioritized human contact, informal interactions, and genuine connections for internal branding. In terms of internal communication, both recognized the impact of digital transformation, demonstrating flexibility, and valuing a balance between formal and informal communication. Technological acceptance was crucial for both, with the competence-oriented approach prioritizing innovation and transparency, while the warmth-oriented approach highlighted spontaneous interaction, positive feedback, personal connections, and emotional engagement. Regarding leadership, both valued regular communication from supervisors, support for employees, and bottom-up initiatives, but competence-focused organizations emphasized adapting to changing work environments by providing training sessions, while warmth-focused organizations stressed leadership in fostering connections and supporting employees through pandemic-induced changes. In conclusion, while each employer brand faces similar challenges, they tailored their approach to align with their unique employer value proposition, thereby reinforcing a distinctive employer brand identity within the organization.”* (see pages 32-34, lines 775 – 819)

5. **I am also concerned about the quotes that are proportionally taken from member check, not the respondents. Member check is about confirming the respondents' answers as well as non-response bias.**

Thank you for your valuable suggestion. As a member check is primarily employed to validate results and responses [63], we have removed the original quotes and replaced them with excerpts from the interviews conducted with the 37 respondents.

6. **In the analysis process, as you mentioned that you used Microsoft Word, how to check inter-rater reliability - a measure of the degree of agreement between multiple researchers analyzing the same data? If we are using a qualitative analysis software such as NVivo, the inter-rater reliability can be measured using the Kappa statistic or percent agreement.**

Thank you for this insightful suggestion. Therefore, we have analyzed the transcripts in NVivo. Afterwards, to ensure the reliability of our coding, we conducted an intercoder reliability assessment. Intercoder reliability assesses the agreement among multiple researchers when assigning codes to themes. Following the recommendations of O’Connor and Joffe [62] and Feng [64], we opted for Krippendorf's alpha (Kalpha) as our measure of intercoder reliability. The Kalpha, calculated in SPSS, yielded substantial agreement. This serves as validation for the coding process and demonstrates that the interpretations and assigned codes maintain consistency across multiple researchers, thereby enhancing the overall reliability and credibility of the study. We have added following paragraph in the method section.

*“Next, in the fifth step, we assessed intercoder reliability using the Krippendorf’s alpha (Kalpha) statistic, a commonly employed measure for such evaluations [74]. A random subset of quotes was provided to another team member. Utilizing the coding reference framework, which includes detailed content information for the subthemes (see 'S3_Tab'), the team member assigned codes to the subthemes following the method outlined by O’Connor and Joffe [74]. This process yielded a Kalpha value of .81, signifying substantial agreement. Ongoing discussions and assessments ensured the continued robustness of our analysis. We made the decision to merge 23 subthemes into 16 subthemes, for instance “Reinforcement of internal employer branding” and “Retention and strengthening of employer brand” consolidating them into a subtheme renamed as "Continuous focus on the internal employer brand". In the sixth step, we implemented the subthemes in the results section and elaborated on these subthemes by providing evidence (i.e. quotes from participants) (see ‘S5_Tab’), conducting a member and employee check.”* (See pages 17-18, lines 416 – 428)

7. **Lastly, please be consistent with the citation style and do another round of editing.**

Thank you for highlighting the need for consistency in the citation style. I have addressed this issue and completed another round of editing to enhance the overall quality of the manuscript.

**REVIEWER 2**

1. **I read with great interest your article on internal employer branding during a crisis. I do see the added value of having more in-depth insights in the art and impact of internal employer branding and also during challenging /crisis times. Overall the manuscript reads well, and is logically structured. I appreciate the transparency on the interviewees’ profile in Table 2, and the extent to which the extra 6 interviewees (member check) agreed or not with the results you provided in Table 1.**

Thank you for your thoughtful response. We are pleased to hear that you found our article on internal employer branding during a crisis interesting, valuable, and well-written. Your recognition of the added value in exploring the nuances of internal employer branding, particularly in challenging or crisis times, is truly encouraging. We also take note of your appreciation for the transparency regarding the interviewees' profile in Supplementary Material ‘S1_Tab’ and the insights gained from the member check. We appreciate your positive feedback and will continue to delve into these aspects to provide meaningful insights.

2. **I struggle the most with a lack of clear added value of your study beyond the mere fact that such research is absent, the lack of the viewpoint of internal employees especially given the aim and practical implications that were formulated (now purely based on responses of HR managers), and the lack of more in-depth rich dynamic insights derived from the data (e.g. also distinction between inductively and deductively deduced themes/codes). I will explain these issues hereafter in more detail.**

Thank you for your comprehensive review and the valuable insights into my research. We appreciate your constructive comments, and we want to convey that we fully understand the concerns you have raised. We acknowledge the need for a clearer presentation of the added value of my study, and we are committed to addressing this concern. We reframed the introduction and aims of the study, and these changes are explicitly outlined in comment 3a and 3b.

Additionally, your observation regarding the absence of perspectives from internal employees and the need for more dynamic insights has been duly noted. In response, we have taken several steps in the subsequent research. We conducted new and in-depth analyses in NVivo, performing a contextual analysis, considering different types of employer brands, and we presented in the supporting information the codes and themes deduced through both inductive and deductive approaches (see ‘S3_Tab’). Additionally, to incorporate the viewpoints of internal employees, we conducted interviews with six employees (= employee check) from organizations that participated in the original interviews. Next, we briefly integrated and discussed their insights in the results section, although it was not the central focus of our study. Nevertheless, we acknowledge the importance of exploring the employee perspective to achieve a more comprehensive understanding. Our main interest lies in obtaining the strategic perspective of HR managers regarding internal employer branding during disruptive events. We have addressed this issue more explicitly in comments 3a, 3b, and 6.

3. **Added value of your research and unclarity in research focus:**
   a) **In introduction it remains unclear why we would need research on internal branding strategies in crisis beyond the mere fact that there is no such research. A stronger rationale is needed as for why we expect current research findings on internal employer branding during stable times would not hold in case of crisis or more unstable periods. Maybe an in-depth look at changes in preferred work values or needs of employees might help as perspective to frame it why it would or could look different in unstable times for HR managers on how to make work of internal employer branding.** **On page 6, lines 129-132 idea of togetherness as more important during crisis can be a starting point, but needs more elaboration. Same goes for touchpoint of leadership on page 7 (lines 157 -162), you mention importance of flexibility by leader and you just name adopting appropriate strategies but any in-depth elaboration lacks of which internal employer branding strategies might then be needed in crisis more or less and why in the sense of likely impact on internal employees’ motivation, satisfaction, ...**

Thank you for your valuable feedback. We appreciated your thoughtful comments aimed at enhancing the clarity and depth of our study. Recognizing the need for a clear rationale to justify research during unstable times, we have refined the introduction and discussion accordingly. In response to your comments, we refined the introduction to explain why research during unstable times is needed.

*“Since the expression ‘war for talent’ suggested by McKinsey, organizations are applying different approaches to compete for talent. Literature has proposed employer branding as an effective organizational strategy to win the ‘war’ by attracting and retaining talented employees [1]. Employer branding aims to differentiate the organization, internally and externally, through its unique employment experience and attractiveness as an employer [2, 3]. To enhance attractiveness, creating and promoting a strong employer within the organization (i.e. internal employer branding) and externally promoting the employer brand through recruitment strategies (i.e. external employer branding) are crucial elements.*

*Research has extensively studied the external promotion of the employer image. Internal employer branding, however, has received little attention compared to external employer branding [4]. Backhaus and Tikoo [5] described internal employer branding as a strategy that creates, promotes, and delivers a distinctive and attractive image as an employer (i.e., employer brand) to employees. To achieve an effective internal employer branding approach, the employer brand has to be present in each aspect of the organization [6]. Hence, employees should experience the internal employer brand through multiple touch-points, such as internal communication and leadership [7, 8]. These aspects, already established as important during stable times, form integral components of an effective internal employer branding strategy. Prior research suggests that a strong internal employer brand relates positively to employees’ attitudes and reduces turnover intentions [9, 10]. However, previous studies typically focused on employee perceptions, largely ignoring the crucial role that HR managers or employer brand managers play in crafting and implementing internal employer branding strategies [11]. Hence, we know little on how strategic decisions with regard to internal employer branding are made and implemented.*

*Furthermore, to extend our knowledge of internal employer branding, scholars like Lievens and Slaughter [12] advocate for exploring the employer brand amid unstable times, emphasizing that current literature predominantly addresses stable environments. This imperative gains significance in an era where crises, fueled by factors such as the rise of social media and accelerated media flows, occur more frequently, amplifying threats to reputations [13, 14]. During a crisis, the employer brand can be vulnerable to damage and negative influences [15]. Crises intensify the pressure on organizations to make strategic decisions. These decisions impacts the immediate well-being of employees that experience enhanced levels of frustration, uncertainty and need for information during a crisis [16]. Additionally, during crises, strategic decisions can influence employees' perceptions of the employer brand, which may prompt questions about the alignment between the organization’s stated employer brand values and the actions taken during challenging circumstances. For instance, if an organization claims to be a warm and supportive employer, will this promise hold on when things get tough and layoffs may seem necessary? Moreover, is the employer value proposition still relevant during a crisis or do certain other aspects become more important, such as creating more togetherness among employees or focusing on the operational functioning? Furthermore, some employer branding practices might become difficult or even impossible in the midst of a crisis, when resources might be lacking or social media unavailable. Navigating this delicate balance between addressing immediate operational needs and preserving the long-term reputation of the employer brand might pose significant challenges for organizations and HR managers are likely to play a crucial role in shaping the internal employer brand during such transformative events.*

*The COVID-19 pandemic, with its unprecedented disruptive nature and its impact on employees’ careers and well-being [17, 18] presents a unique opportunity to delve into the intricacies of internal employer branding during a crisis. This crisis not only necessitated organizational adaptations but also left an indelible impact on employees [19, 20]. During the pandemic, organizations were forced to adopt new working routines and policies to ensure business continuity. At the same time, these restrictions and working routines threatened employees’ well-being due to the inability to separate work-private life, the feeling of job insecurity, and loneliness [17, 18, 21]. It created an environment where employees experienced uncertainties, changing work patterns, and feelings of isolation. Thus, the COVID-19 pandemic, with its challenges, has been a disruption that overturned existing working arrangements [22] and threatened employees’ well-being [23]. Therefore, examining internal employer branding during the COVID-19 pandemic provides a suitable context to understand how HR managers navigate challenges and safeguard their organization’s employer brand in times of crises.*

*This study aims to deepen our understanding of how internal employer branding is influenced by disruptive events, specifically exploring the strategic decisions made and implemented by HR managers during the pandemic. We also aim to investigate the role of the key internal employer branding touchpoints internal communication and leadership during a crisis, as they may be vital for adapting employees to new conditions and challenges [24]. For instance, the provision of emotional support, empathy, a relationship-oriented communication, and supportive leadership might enhance a sense of togetherness, trust, and support among employees, potentially fostering commitment to the employer brand during the pandemic.*

*To this end, in-depth interviews were conducted with 37 HR managers from 37 Belgian organizations that varied in sector and size. To validate our findings, we conducted a member check with six other HR managers from different organizations and an employee check with six employees from the organizations that participated in the study. On a theoretical level, our study makes at least two significant contributions. First, we shed light on how HR or employer brand managers make and implement strategic decisions regarding internal employer branding. We thus extend prior research that has mostly looked at employer brand perceptions held by employees and answer the call for more organization-level, strategic HRM research. Second, our research explores the dynamics of internal employer branding during crises, offering insights into the challenges and opportunities they may create and how HR managers respond to them. These insights complement prior research conducted in stable times. Regarding practical implications, our findings are useful for HR managers wanting to learn from their colleagues and how they have handled internal employer branding during disruptive events. It can make them more aware of the strategic considerations involved and their own decision-making process*.*” (see page 2-6, lines 44 – 126)*

Moreover, we have enhanced the discussion to elucidate our contribution to the research. The following paragraphs have been revised in the manuscript:

*“Thirdly, this study underscores the critical role of HR managers as central figures in navigating internal employer branding during crises, with a specific focus on the COVID-19 pandemic [11]. Theoretical implications of these findings provide insights into the decision-making processes of HR managers concerning internal employer branding during crises. The research underscores the strategic engagement of HR managers, their innovative solutions, and their efforts to uphold the employer brand in challenging times.” (see page 38, lines 931 – 936)*

b) **After introduction and literature it remains unclear what the exact research focus and question is. Is it about which internal branding strategies are adopted by HR managers during crisis or what impact one might have on employees of adopting same or changed internal branding strategies in terms of internal communication/leadership? If you want to contribute with your research to practice by being able to guide organizations in handling their internal employer branding during future crisis (end of introduction), I do not understand how you can do that validly by only questioning and studying the view point of HR managers. At least we would need to have an idea of the impact of certain adopted internal employer branding strategies by studying then the voice and perspective of employees themselves (what were there needs, changed work anchors or work values, how did they experience the adopted internal employer branding during crisis?) . Also at the end of the literature section on ‘ employer branding’ (line 90-92) you repeat that research on internal employer branding is scarce and that therefore you will focus on exploring internal employer branding during unstable times, but why this is relevant and e.g. hence different from stable times remains untouched.**

Thank you for your insightful comment. Recognizing the limitations of focusing solely on HR managers, we reframed our research goal in the introduction (see comment 3a), aims of the study, and discussion. Our study aims to uncover HR managers' internal branding strategies during unstable times, specifically examining internal communication and leadership. We delved into challenges and opportunities during the COVID-19 pandemic, deepening understanding of how disruptions influence internal employer branding.

“*Amidst the dynamic landscape of disruptive events, marked by an anticipated increase in the frequency of crises [21], there exists a noticeable gap in the exploration of challenges and opportunities pertaining to internal employer branding, internal communication, and leadership. The existing body of research has predominantly focused on stable times and employee perceptions. In response to the call of Lievens and Slaughter [12] to investigate employer branding in unstable times, we explore how HR managers make and implement strategic decisions regarding internal employer branding and its associated touchpoints during a crisis. How HR managers approach the intricacies of internal employer branding during tumultuous times, and the inherent difficulty of adhering to established employer brand values amidst crisis-induced shifts, remains insufficiently understood. The uncertainty and pressure of a crisis may necessitate strategic decisions that could potentially deviate from the conventional employer branding focus. Questions arise about the feasibility of upholding a warm and supportive employer image during times of organizational upheaval, such as layoffs or operational adjustments. Hence, the exploration of internal employer branding becomes imperative, offering insights into the nuanced strategies employed by HR managers and the potential shifts in focus within employer branding practices.*

*Therefore, it is our goal to provide insight into how HR managers perceived and experienced the impact of the COVID-19 pandemic on internal employer branding and the two touchpoints internal communication and leadership. As the pandemic yielded negative implications for organizations’ business continuity, we can expect a similar challenging impact on internal employer branding, internal communication, and leadership. Therefore, the following research question was formed:*

*Research Question 1: What challenges have HR managers experienced during the COVID-19* *pandemic regarding internal employer branding and internal employer branding touch-points (i.e. internal communication and leadership)?*

*Furthermore, in times of crisis, managers have an exceptional opportunity to learn or refine strategic decisions, which may have not been straightforward before. According to Akkermans et al., [17], employees or organizations who experience a critical negative workplace event are more likely to engage in improvisation behaviors such as taking advantage of opportunities and “thinking outside the box” [57]. Dealing with these challenges, HR managers might take a different and more creative employer branding approach to address changing employee needs and concerns. Accordingly, we can expect that possible opportunities may arise during critical events for internal employer branding and its touch-points. As such, the following research question was formed:*

*Research Question 2: What opportunities have HR managers experienced during the COVID-19 pandemic regarding internal employer branding and internal employer branding touch-points (i.e. internal communication and leadership)?”* (see pages 10-12, lines 244-281)

Furthermore, our deliberate focus on HR managers for employer branding stems from their central role in both normal and crisis situations. While external employer branding is well-explored, the internal aspect crucial for employee attitudes is understudied. HR managers, often overlooked, actively contribute to overarching strategies and execution. Recognizing their pivotal role, our research aligns with the insights presented by Edlinger [11]. By delving into the perspectives of HR managers, we strive for a comprehensive understanding of the employer branding process, exploring how crises impact organizations and the alignment between employer brand values and actions. This focus on HR managers is detailed in the introduction and discussion, but further elucidated in the method section, particularly in the 'Data Collection' subsection.

*“As the perspective of HR managers about internal employer branding during unstable times lacks clear understanding and knowledge [11, 12], a qualitative approach was adopted. Qualitative research - due to its innate explorative nature - allows for more detailed insights into a topic, as it builds upon the perceived realities of its target population [58]. It enables us to capture the nuances of this relatively unexplored topic. Therefore, this study specifically delved into the perceptions and experiences of HR managers regarding their internal employer branding strategies and practices during the pandemic.*

*Our participant selection involved* *purposeful and maximum variation sampling [59]. Purposeful sampling was utilized to identify and choose participants whose characteristics align with the focus of our investigation [60]. It is particularly useful when the researcher intends to recruit participants who possess specific information based on their professional experiences [61, 62]. For the current study, we intended to recruit participants that were responsible for (internal) employer branding. Furthermore, since the objective was to enhance our comprehension of the phenomenon [63, 64], we intentionally sought diversity through maximum variation. Consequently, we selected organizations that maximally differed on two conditions that may influence employer branding activities, namely organization size and sector [65, 66].*

*To facilitate open sharing of experiences and perceptions, including potential negative views or personal accounts, we chose individual semi-structured interviews to collect the data [67, 68]. The interviews were carried out by two master students in HRM and a doctoral researcher. We trained students’ interview techniques before and during the interviews. Using our network, we contacted organizations via email in which we specified the topics to be addressed. The interviews were conducted through video calls, as teleworking was the norm at that time, and were recorded.*

*The interviews were based on insights retrieved from a literature review. Before we started the interview, we made sure that there was a common understanding of the concept of internal employer branding by asking what they understood regarding this topic and providing a definition of this concept. The interview guide covered three main categories: (a) internal employer branding, (b) internal communication about employer branding, and (c) leadership in function of employer branding (see ‘S3_Tab’). Furthermore, in line with guidelines, the initial interview questions were regularly updated based on the presented information by HR managers during this data-collection process [69]. We applied the principles of data saturation and information redundancy. Our data collection ended when additional interviews did not bring new information. This was achieved after about 30 interviews, but seven control interviews were still conducted to be sure. Participants were instructed to provide honest answers based on their experiences during the COVID-19 pandemic.”* (see pages 13-15, lines 320– 356)

4. **Clarity on distinction between internal brand and internal employer brand**
a) **The authors clearly describe in beginning of manuscript the difference between internal brand and internal employer brand (lines 101-103). However, in literature but especially in the results there are many statements that point toward internal brand in terms of how employees embrace it and translate it in how they interact with customers, how they do their work instead of reflecting what makes the culture, the work practices, the employer in general unique and worth to stay with in a motivated way as employee. Internal brand instead of internal employer brand: For example line 150 in literature. For example in results lines 325-329.**

Thank you for this suggestion. In the initial sections of our manuscript, we indeed delineated the distinction between internal brand and internal employer brand. Nevertheless, we acknowledge the valid concern raised by the reviewer regarding the potential ambiguity in the literature and results sections. We have revisited the literature section and conducted a careful review of our language use. Recognizing the importance of clarity, we have refined our expressions in the theoretical background to ensure that the focus remains on internal employer branding, emphasizing aspects such as organizational culture, work practices, and the unique attributes of the employer that contribute to fostering employee retention. Following paragraphs were included.

“*Internal communication, as defined by Kalla [41], encompasses all formal and informal exchanges of information within an organization, playing a crucial role in daily and strategic activities [42]. Recognized as pivotal, it is fundamental for successful employer brand development and management, ensuring consistent perceptions among internal and external stakeholders [43]. Internal communication about the employer brand, managed by HR managers, serves as a tool for conveying employment advantages, sharing core values, aligning employee behavior with the employer brand, and fostering commitment and loyalty [6, 44, 45, 46].*

*Moreover, it aids in developing committed and loyal employees who identify with the employer brand values [36]. According to Itam et al., [47], internal communication reduces information asymmetry among current employees by sharing the organization’s mission and values. HR managers view internal communication as collaborative employer brand crafting [48], facilitating the exchange of information and unique values related to the employer brand [42]. It plays a pivotal role in shaping employee attitudes toward employer branding [7, 37]. Effective communication is thus a key factor in successful employer brand development and management, ensuring uniform perceptions among internal and external audiences and employees’ attitudes toward the employer brand during stable times.*

*In times of crisis, organizations face the challenge of reassessing traditional approaches that may prove inadequate under the pressures of such situations. The COVID-19 pandemic introduces new working practices, particularly the transition to remote and hybrid work, potentially influencing how employees perceive the employer brand. Recognizing the need for a deeper understanding, organizations may consider adopting a communication strategy focused on fostering relationships, unity, and a sense of togetherness, aiming to ensure employee satisfaction during a crisis. Nelke [49] shows that this can help to increase trust among employees. Moreover, according to Sinčić Ćorić and Špoljarić [42], satisfaction with internal communication is important for employees to foster engagement and employee satisfaction. Therefore, to craft an effective internal communication strategy crucial for shaping, understanding, and fostering a positive perception of the employer brand during a disruptive event, HR managers may need to adapt the delivery approaches, modify content, or alter the manner in which it is presented to employees.*” (see pages 8-9, lines 179 – 209)

*“Supervisors can influence, motivate, and enable employees to achieve a specific goal [50]. Leadership concerning employer branding includes all the efforts of leaders to disseminate the employer brand principles to employees, which entails showing the work culture and unique work practices. HR managers rely on supervisors to convey the employer brand to employees, forming a crucial partnership in delivering the employer brand to employees. Zeesahn et al., [51] emphasized that organizations should focus on both leadership and internal employer branding to retain and attract employees. Moreover, leadership has a significant part in creating an employer brand climate and establishing an inner atmosphere which impacts employees’ attitudes [52].*

*Barrow and Mosley [53] emphasize supervisors’ pivotal role in developing and communicating the employer brand. For instance, supervisors can actively embody the employer brand by sharing personal experiences that reflect the employer brand values during team meetings. Concurrently, supervisors, as suggested by Biswas and Suar [54], can provide clear directives, like implementing regular team-building activities, to showcase distinctive work practices. These visible leadership actions not only influence how employees perceive the internal employer brand, values, and culture, but can also support them in incorporating these aspects into their daily tasks [54]. For instance, during a daily team meeting, employees can proactively contribute to a warm and collaborative atmosphere by sharing positive experiences related to teamwork or recognizing a colleague's effort. This daily practice aligns with the employer brand's emphasis on collaboration and fosters a positive workplace culture. Therefore, to achieve a strong internal employer brand, supervisors have to actively “live the employer brand”, and display the employer brand in their interactions with employees.*

*Nevertheless, during a disruptive event, supervisors can rise to the challenge while other supervisors will struggle to manage the crisis [55]. Supervisors are relying on their instincts and insights, supported by HR managers, to ensure their employees feel supported. Dirani et al., [55] concluded that when employees face challenges in a new work environment, supervisors must become more flexible to adjust their employees to the new altered work environment. Moreover, supervisors may need to implement targeted approaches to address the likely impact of a crisis on employees' motivation, satisfaction, and overall well-being. This could involve strategies such as transparent communication, empathetic leadership, and tailored support mechanisms [56]. As such, to foster employer brand-aligned leadership during turbulent times, it is crucial to explore how HR managers guide supervisors in navigating challenges and promoting the employer brand among employees*.” (see pages 9-10, lines 210 – 243)

Furthermore, we have conducted a new data analysis using NVivo, with a focus on gaining a comprehensive understanding of organizational culture, unique values, and work experiences. Our approach has been centered on these aspects, and we have adjusted our results section accordingly. Literature underscores the impact of a robust internal employer brand on employee support and organizational goals. While external employer branding attracts talents, internal employer branding encourages existing employees to endorse and contribute to the organization's objectives. Managing the internal employer brand fosters employee engagement and translates into customer interactions, making it an integral part of the overall process [31, 34, 66].

b) **The results with regard to more supportive leadership point at some places to ‘when supervising and supporting employees to radiate the employer brand’, but the question presents itself is this about radiation to external stakeholders (hence internal brand) or just in general a more people-centered leadership style regardless of the link with bringing alive the internal employer brand. More in-depth elaboration on these results with how then precisely this affects managing the internal employer brand could remove these doubts. In the next point some more examples will be shared where the richness of the data is not translated in the results.**

Thank you for your feedback. To address the concerns about supportive leadership, we have reanalyzed and clarified in the results section with specific instances. This enhancement illustrates how supportive leadership impacts the internal employer brand through new initiatives and guidance, such as effectively communicating the work culture, values, and the work environment.

*“Respondents emphasized the pivotal role of supervisors showcasing a more people-centered leadership style, especially during the pandemic. HR managers advised supervisors to transition towards a more understanding and supportive leadership style when delivering the employer brand and to trust employees to implement the employer brand accurately. During the pandemic, supervisors were directed to emphasize people skills in delivering the employer brand, as highlighted by the 12 HR managers. This shift was reflected in their active attention to team members, going beyond traditional methods. Notable initiatives included personalized virtual team-building sessions, like online coffee meet-ups and fitness challenges, aimed at conveying organizational values and fostering a cohesive internal environment.*

*HR managers highlighted the importance of supervisors understanding employee needs, fostering a culture of mentorship, and playing a crucial role in providing support during challenging times. This involvement was seen as integral to reinforcing the organization's commitment to employee welfare and the employer brand. Recognizing the significance of supervisors prioritizing their teams, HR managers highlighted its positive impact on conveying an image aligned with employees' values.*

*The participants noted that supervisors, in response, organized enjoyable initiatives to enhance the internal delivery of the employer brand. This proactive approach, aligned with a people-centered leadership style, demonstrated a commitment to workforce growth and cohesion. Supervisors actively contributed to shaping the internal employer brand by providing support and fostering a skilled and closely-knit team. Despite these positive shifts, HR managers reported that the adoption of a different mode of delivering the employer brand was not uniformly evident for every supervisor. An HR manager mentioned:*

*Some supervisors had a more controlling approach when supervising and supporting employees on radiating the employer brand. During the pandemic, supervisors were forced to adapt their behavior – as employees were working from home and direct supervision was not possible. This was a challenge for supervisors – as they were forced to develop people skills and transform their leadership style. (male, large organization, hospitality)”* (see pages 31-32, lines 742– 771)

5. **Lack of in-depth insights, more richness needed**
   a) **In subtheme 2b the result is presented that the internal employer brand is communicated more in a warm way (line 416). It is nice that the authors also name a case in which misinterpretations on internal employer brand could be solved with short wording. Then an in-depth analysis could reveal if this short style is to reconcile with the warmth? Or is this not an appropriate question as both strategies may relate to different types of internal employer brands. It would be more nuanced to allow the reader to have some basic idea of the core elements of the internal employer brands that were studies along the 36 respondents. We could argue that internal employer brands being higher on warmth and togetherness also before crisis might have experienced different challenges and opportunities versus internal employer brands being focused on e.g. efficiency or competency or … This richness as well as the dynamic perspective can be much more developed and is needed to more validly understand any contextually conditions that might have affected the results for example with regard to focusing more on togetherness, on warmth, ... Based on the definition of internal employer brand one could wonder if there are any instrumental or other symbolic factors that came to the surface in terms of posing more challenges or opportunities.**

Thank you for the feedback and suggestion for an in-depth analysis. We revisited analyses in NVivo, particularly on the relationship between warm communication and brief wording. While investigating if brief wordings are linked to communication warmth, limited responses prevent a conclusive finding. However, we explored challenges and opportunities for different employer brands, leading to adjustments in the results section and discussion, introducing a new subsection in the results titled "Exploring the Context: Warm vs Competent Employer Brands."

“*To incorporate contextual elements, we investigated whether distinct types of employer brands—warm versus competent, encountered different challenges and opportunities. We initiated a contextual analysis by first soliciting descriptions of internal employer branding and the content of employer brands during the interviews. Organizations that articulated a familial, warm, or social emphasis in their descriptions were categorized as warm employer brands. Conversely, those highlighting aspects such as training opportunities, employee growth, and competence development were classified as competent employer brands. Out of the 37 organizations analyzed, we successfully categorized 28, identifying 11 as warm employer brands and 17 as competent employer brands. This categorization formed the foundation for further exploration, allowing us to investigate whether and how these distinct employer brand types encountered unique challenges or opportunities amidst the pandemic.*

***Content***

*Examining the content of these two types of the employer brand, it is noteworthy that challenges across the two types of employer brands appear largely similar. However, the key difference lies in their content. Competence-oriented employer brands prioritize formality, competencies, and efficiency within a professional framework. They focus on efficiency, innovation, and digital transition. In contrast, warmth-oriented brands emphasize personal connections, care, well-being, employee recognition, and a positive atmosphere during the pandemic. They present well-being as part of a broader positive branding strategy, utilizing symbolic language and digital communication. The communication style is informal, fostering an open atmosphere and focusing on individual well-being to enhance team culture and relationships. Despite facing common challenges, each approach customizes its employer brand transfer and communication style according to its unique employer value proposition, reinforcing its distinct employer brand identity.*

***Approach***

*Both competence-oriented and warmth-oriented employer brands showed a strong commitment to internal branding during the pandemic, emphasizing on engagement and employer brand values. Competency-focused organizations strategically addressed the challenges, prioritizing innovation, and preserving organizational culture. In contrast, warmth-focused organizations prioritized human contact, informal interactions, and genuine connections for internal branding. In terms of internal communication, both recognized the impact of digital transformation, demonstrating flexibility, and valuing a balance between formal and informal communication. Technological acceptance was crucial for both, with the competence-oriented approach prioritizing innovation and transparency, while the warmth-oriented approach highlighted spontaneous interaction, positive feedback, personal connections, and emotional engagement. Regarding leadership, both valued regular communication from supervisors, support for employees, and bottom-up initiatives, but competence-focused organizations emphasized adapting to changing work environments by providing training sessions, while warmth-focused organizations stressed leadership in fostering connections and supporting employees through pandemic-induced changes. In conclusion, while each employer brand faces similar challenges, they tailored their approach to align with their unique employer value proposition, thereby reinforcing a distinctive employer brand identity within the organization*.” (see pages 32-34, lines 775 – 819)

We have altered the discussion accordingly:

*“Finally, our results showed that strategies and opportunities adopted to navigate the challenges during the pandemic varied based on their employer brand orientation, specifically warm or competent [12, 67]. Both orientations encountered similar difficulties related to new strategies, onboarding, remote work, and maintaining a connection with the employer brand. Despite these shared challenges, competence-focused organizations strategically approached these issues, emphasizing innovation and the preservation of organizational culture. In contrast, warmth-focused organizations prioritized human contact and informal interactions, emphasizing the importance of genuine connections for internal employer branding. This observation may suggest, though it requires further investigation, that the employer brand plays a pivotal role in shaping distinct priorities and approaches when addressing internal employer branding in times of crisis. Moreover, this study indicates organizations’ commitment to living the employer brand, staying true to it, and avoiding radical changes during a disruptive event.”* (see pages 37-38, lines 909 – 921)

*“Lastly, this research suggests based on the interviews that different types of employer brands (warm vs. competent) face similar challenges during a crisis, but may adopt distinct strategies. The employer brand or employer value proposition serves as a crucial boundary condition during the pandemic, shaping the organization's response to external uncertainties and internal dynamics”* (see pages 39, lines 952 – 956)

*“Furthermore, this study reveals that different types of employer brands, specifically those characterized as warm versus competent, encounter similar challenges amid crises but may adopt unique strategies based on their employer value proposition. Different theoretical frameworks, such as Instrumental-Symbolic Framework [68], could be used to incorporate a deeper understanding of how crisis events impact diverse employer brand orientations. Future research could also delve into the mechanisms through which organizations strategically align their internal branding efforts in response to crises, shedding light on the dynamic interplay between external uncertainties and internal organizational dynamics. Unpacking the influence of these elements on organizational responses to crises remains an essential area for exploration. Future studies could investigate how variations in employer brand messaging and value propositions shape decision-making processes, internal communication strategies, and overall resilience during challenging times. ”* (see pages 41-42, lines 1013 – 1024)

b) **It would enhance the internal validity if themes/codes could be separated that were deductively versus inductively derived, e.g. could be added to Table 1. Now the results read as if only the lens of prior literature on internal communication and leadership as essential touchpoints were followed to analyze the transcriptions with no openness toward new findings, like other touchpoints that might have become more essential just because of the crisis (e.g. any instrumental attributes) beyond communication or leadership, or same touchpoint but managed differently e.g. like you bring in results in terms of bringing more warmth in communication and leadership (lines 416 and line 430, 433).**

We appreciated the insightful suggestion to enhance internal validity by distinguishing themes and codes derived deductively versus inductively. Consequently, we have included this information in supporting information 'S2_Tab,' explicitly specifying the deductive or inductive origin of each theme. Additionally, in supporting information 'S3_Tab,' we have detailed our coding process, providing a step-by-step progression, including when each theme emerged or was renamed—prior to coding, after 15 interviews, after 37 interviews, and post intercoder reliability calculation and team discussion. Furthermore, we included the following paragraph in the method section.

“*The thematic analysis consists of six phases and was performed using NVivo 8. In the first phase, we familiarized ourselves with our data by transcribing and (re-)reading the data. During this step, we marked sentences and wrote down our first impressions. In the second phase, prior to initiating the coding process, we pre-established nine themes deductively, including categories such as "policy," "policy challenges," and "policy opportunities". Subsequently, initial codes were generated based on patterns that emerged from the collected data. During the initial coding, we were open to new insights and contradictions and took into account the organizational context. The first author was mainly responsible for the coding, but the coding and themes were frequently discussed with the other authors, and issues were resolved in consensus. In the third phase, following the coding of 15 interviews, our initial focus involved identifying new subthemes within our coding. Themes like "loss of contact with the employer," "digitalization", and "new leadership skills" surfaced during this phase. Upon completing the coding for all interviews, we conducted an analysis by shifting our attention to a broader level of themes. This involved identifying overarching themes, such as various types of employer brands, organizational size, and sector. We built a thematic map of the codes and reflected on the relationship between these themes. At the end of this step, we identified three main themes (e.g. “internal employer branding”, “internal communication”, and “leadership”) and six themes (“policy challenges”, “policy opportunities”, “internal communication challenges”, “internal communication opportunities”, “leadership challenges”, and “leadership opportunities). As thematic analyses allow for the emergence of inductive themes, we identified a total of 24 inductive subthemes within the deductive (main)themes. These included topics like "flexibility and well-being," "retention and strengthening of employer brand values," "loss of informal communication and connectedness," "message," and more. In the fourth step, we reviewed, refined, and renamed the themes and looked for overlapping themes. While retaining the three main themes and six themes, we settled on 23 subthemes. Notably, we discussed and opted to merge the themes "leadership visibility" and "fostering communication" into a single theme, named “Leadership visibility through communication”. Next, in the fifth step, we assessed intercoder reliability using the Krippendorf’s alpha (Kalpha) statistic, a commonly employed measure for such evaluations [74]. A random subset of quotes was provided to another team member. Utilizing the coding reference framework, which includes detailed content information for the subthemes (see 'S3_Tab'), the team member assigned codes to the subthemes following the method outlined by O’Connor and Joffe [74]. This process yielded a Kalpha value of .81, signifying substantial agreement. Ongoing discussions and assessments ensured the continued robustness of our analysis. We made the decision to merge 23 subthemes into 16 subthemes, for instance “Reinforcement of internal employer branding” and “Retention and strengthening of employer brand” consolidating them into a subtheme renamed as "Continuous focus on the internal employer brand". In the sixth step, we implemented the subthemes in the results section and elaborated on these subthemes by providing evidence (i.e. quotes from participants) (see ‘S5_Tab’), conducting a member and employee check.”* (see pages 16 – 18, lines 389 – 428)

6. **View of employees needed: One of the results is that HR managers identified that employees had difficulties in recognizing the employer brand (line 293/294). But the question presents itself if this is problematic during crisis and why so. So this result could gain much more relevance if it could be linked to the impact on the employees and why it matters that employees had difficulties in recognizing the employer brand. Were they more easily seduced to leave or did it affect the culture in a negative way or …. Now the results read as what HR managers did and struggled with in terms of internal employer branding efforts, and that they attached importance to internal employer brand even in crisis. While the latter is a useful finding, the question that needs to be equally solved is then ‘and does it matter in times of crisis to focus or re-focus on internal employer brand and to do it differently with e.g. more warmth like you describe. In the limitations section it is acknowledged that the adoption of certain internal employer branding strategies and its evaluation might differ in the perspective of HR managers and employees, but simply adding this as a limitation does not overcome the impossibility to make claims on guiding organizations on internal employer branding during crisis if you have not tested how the adoption was perceived in the first place by employees (was effort noticed) and secondly how was it evaluated (was effort beneficial for certain outcomes like retention) or were there other needs of employees that were or were not addressed by the internal employer branding efforts during crisis period which might reveal other meaningful internal employer brand challenges during crisis? Therefore, additional data collection is really needed to embrace the experience of employees on the efforts done by HR managers. The latter, the sole focus of the study now, risks missing valuable insights on how the internal employer brand efforts pays off, and is too descriptive in nature like it is brought now in this version of the paper. In-depth rich contextual sensitive and dynamic perspectives can add more valid contributions to the internal employer branding literature than what is currently available in the paper.**

Thank you for this suggestion. While our primary focus remains on capturing the experiences and practices of HR managers in this study, we acknowledge the importance of incorporating more contextual sensitivity and dynamic perspectives. To address this, we conducted a follow-up study, specifically an employee check. In this phase, we interviewed six employees, sharing our findings and seeking their opinions on HR perspectives and their impacts. The outcomes of these employee interviews have been integrated into the dedicated section titled "Employee Check” in method, result and discussion section.

Method:

*“Finally, we conducted employee checks to ensure that the conclusions derived from the interviews were experiences that were recognizable to the employee target group [71]. This allowed us to verify whether members of the target group of internal employer branding agreed with our findings. Moreover, this enabled us to supplement our results with their input. As such, in December 2023, we carried out an employee check to authenticate and confirm the research findings from the employees' standpoint. We reached out to all 37 organizations and conducted interviews with six employees, each lasting between 25 and 35 minutes. During these semi-structured interviews, we inquired the participants about their experiences with the strategies and practices related to employer branding that were implemented during the COVID-19 pandemic, using the 16 statements and results as a basis for discussion. Prior to posing these questions, we ensured that employees had a clear understanding of the concept of employer branding. Subsequently, employees were given the opportunity to elaborate on these statements. Consult 'S1_Tab' for participant details*.” (see pages 15 – 16, lines 367-380)

Results:

*“To gain insights from employees, we conducted a check involving individuals from the same organizations as those interviewed. Overall, employees validated the findings pertaining to internal employer branding, internal communication, and leadership (see 'S4_Tab'). Participants highlighted challenges in perceiving the employer brand due to physical distance, resulting in diminished connection and commitment. Traditional methods of communicating the employer brand to current employees encountered pandemic-related obstacles, as indicated by HR managers and corroborated by employees. The employee check additionally observed a shift toward warmth and care in employer brand communication.*

*HR managers noted a decline in bottom-up communication, potentially leading to misinterpretations, a sentiment supported by the employee check. Furthermore, HR managers discussed difficulties in monitoring employees and encouraged supervisors to regularly check in with their teams, a point substantiated by the employee check. Employees reported an increase in their interactions with supervisors.*

*In addressing the need for a different leadership style during the pandemic, HR managers emphasized coaching and support in conveying the internal employer brand. However, many employees did not perceive this as supervisors delivering the employer brand more frequently; instead, they acknowledged increased support through regular check-ins, assistance, and flexibility. This suggests that while positively influencing the employee experience, supervisors' actions may not be explicitly seen as a direct communication or embodiment of the employer brand. Employees were cognizant of training sessions for supervisors.”* (see pages 34-35, lines 836 – 857)

Discussion:

“*Finally, recognizing the significance of supervisors during unstable times, organizations may consider assigning additional responsibilities to them, including the task of explaining the employer brand to new employees and addressing misinterpretations. This allows HR managers to focus more on the strategic aspects of internal employer branding. Providing support to supervisors in developing interpersonal skills becomes crucial during turbulent periods, emphasizing the need for a shift towards interpersonal skills over technical skills when conveying the employer brand. Although HR managers instructed supervisors to radiate the employer brand more, findings from the employee check revealed that participants did not necessarily perceive supervisors as embodying the employer brand. However, it is noteworthy that while supervisors might not have been seen as direct embodiments of the employer brand, participants expressed appreciation for their consistent check-ins, flexibility, and support. This suggests that, even if not explicitly recognized for employer brand representation, supervisors played a crucial role in fostering positive employee experiences through their supportive actions.”* (see pages 40-41, lines 980 – 993)

“*Although we conducted an employee check to explore the perspective of the employees, we mainly investigated the perspective of the HR manager. However, due to the virtual setting of most participating organizations and the physical distance between HR managers and employees, the perceptions of HR managers could potentially be clouded or the impact on employees could be perceived differently by the respondents. Nishii et al., [69] highlighted the potential discrepancy between HR managers’ actual practices and employees’ perceptions of these actions. Therefore, future research can take on a multilevel approach and investigate the perspective of HR managers, employees, and supervisors and compare these perceptions. This approach would provide a more nuanced and holistic view of internal employer branding practices, allowing for a deeper exploration of the dynamics between organizational strategies and their perceived impact on different levels within the workforce.*” (see pages 42-43, lines 1035 – 1045)

7. **Minor issues**
   a) **In Table 2 IN-8 is labeled with age of 14, that seems a typo as in line 213 minimum age is 25.**

Thank you for your feedback. We have addressed the issue you pointed out regarding Table 2 IN-8 (see ‘S1_Tab’). It seems there was a typo in the age label, which we have corrected. In line 213, the minimum age is correctly specified as 35 instead of 14.

b) **Some spacing issues in sentences resulting in words that stick together like on lines 62 guide organizations, or line 75 fits their or line 569 on disseminating.**

Thank you for bringing up the spacing issues. We have reviewed the manuscript and addressed the concerns you raised. We have adjusted the spacing in sentences on lines 62, 75, and 569 to ensure that words are appropriately separated.

8. **I wish the authors good luck with their further work on internal employer branding and look forward to see more rich and nuanced results.**

Thank you for your many developmental suggestions throughout this review. We truly appreciate your constructive feedback and hope that the quality of our work is improved based on your insights.

**REFERENCES**

1.	Reis I, Sousa MJ, Dionísio A. Employer branding as a talent management tool: A systematic literature revision. Sustainability. 2021;13(19):1-22.

2.	Edwards MR. An integrative review of employer branding and OB theory. Personnel Review. 2010;39(1):5-23.

3.	Jonsen K, Point S, Kelan EK, Grieble A. Diversity and inclusion branding: a five-country comparison of corporate websites. The International Journal of Human Resource Management. 2019;32(3):616-49.

4.	Andersson R. Employees as ambassadors: embracing new role expectations and coping with identity-tensions. Corporate Communications: An International Journal. 2019;24(4):702-16.

5.	Backhaus K, Tikoo S. Conceptualizing and researching employer branding. Career Development International. 2004;9(5):501-17.

6.	Gilani H, Cunningham L. Employer branding and its influence on employee retention: A literature review. The Marketing Review. 2017;17(2):239-56.

7.	Verčič AT. The impact of employee engagement, organisational support and employer branding on internal communication satisfaction. Public Relations Review. 2021;47(1):1-7.

8.	Sahu S, Pathardikar A, Kumar A. Transformational leadership and turnover: Mediating effects of employee engagement, employer branding, and psychological attachment Leadership & Organization Development Journal. 2018;39(1):82-99.

9.	Kashyap V, Verma N. Linking dimensions of employer branding and turnover intentions. International Journal of Organizational Analysis. 2018;26(2):282-95.

10.	Ta'Amnha M. Institutionalizing the Employer Brand in Entrepreneurial Enterprises. International Journal of Economics and Financial Issues. 2020;10(6):183-93.

11.	Edlinger G. Employer brand management as boundary‐work: A grounded theory analysis of employer brand managers' narrative accounts. Human resource management journal. 2015;25(4):443-57.

12.	Lievens F, Slaughter JE. Employer image and employer branding: What we know and what we need to know. Annual Review of Organizational Psychology and Organizational Behavior. 2016;3:407-40.

13.	Singh I, Singh J, Baruah A. Income and employment changes under COVID-19 lockdown: A study of urban Punjab. Millennial Asia. 2020;11(3):391-412.

14.	Zheng B, Liu H, Davison RM. Exploring the relationship between corporate reputation and the public’s crisis communication on social media. Public Relations Review. 2018;44(1):56-64.

15.	Garas SRR, Mahran AFA, Mohamed HMH. Internal corporate branding impact on employees’ brand supporting behaviour. Journal of Product & Brand Management. 2018;27(1):79-95.

16.	Johansen W, Aggerholm HK, Frandsen F. Entering new territory: A study of internal crisis management and crisis communication in organizations. Public Relations Review. 2012;38(2):270-9.

17.	Akkermans J, Richardson J, Kraimer ML. The Covid-19 crisis as a career shock: Implications for careers and vocational behavior. Journal of Vocational Behavior. 2020;119(2020):1-5.

18.	Collings DG, McMackin J, Nyberg AJ, Wright PM. Strategic human resource management and COVID‐19: Emerging challenges and research opportunities. Journal of Management Studies. 2021;31(2021):819-33.

19.	Bailey K, Breslin D. The COVID‐19 pandemic: what can we learn from past research in organizations and management? International Journal of Management Reviews. 2021;23(1):3-6.

20.	Lee SM, Trimi S. Convergence innovation in the digital age and in the COVID-19 pandemic crisis. Journal of Business Research. 2021;123:14-22.

21.	Carnevale JB, Hatak I. Employee adjustment and well-being in the era of COVID-19: Implications for human resource management. Journal of Business Research. 2020;116:183-7.

22.	Kim S, Vaiman V, Sanders K. Strategic human resource management in the era of environmental disruptions. Human Resource Management. 2022;61(3):283-93.

23.	Mihalache M, Mihalache OR. How workplace support for the COVID‐19 pandemic and personality traits affect changes in employees' affective commitment to the organization and job‐related well‐being. Human Resource Management. 2022;61(3):295-314.

24.	Labovich L. How To Protect Your Brand During A Layoff. Jersey City, NJ: Forbes. 2016.

25.	Lievens F, Van Hoye G, Anseel F. Organizational identity and employer image: Towards a unifying framework. British Journal of Management. 2007;18:45-59.

26.	Backhaus KB. An exploration of corporate recruitment descriptions on Monster. com. The Journal of Business Communication. 2004;41(2):115-36.

27.	Barros-Arrieta D, García-Cali E. Internal branding: conceptualization from a literature review and opportunities for future research. Journal of Brand Management. 2021;28(2):133-51.

28.	Theurer CP, Tumasjan A, Welpe IM, Lievens F. Employer branding: a brand equity‐based literature review and research agenda. International Journal of Management Reviews. 2018;20(1):155-79.

29.	Saini GK, Lievens F, Srivastava M. Employer and internal branding research: a bibliometric analysis of 25 years. Journal of Product & Brand Management. 2022;31(8):1196-221.

30.	Mosley RW. Customer experience, organisational culture and the employer brand. Journal of Brand Management. 2007;15(2):123-34.

31.	Staniec I, Kalińska-Kula M. Internal employer branding as a way to improve employee engagement. Problems and Perspectives in Management. 2021;19(3):33-45.

32.	Kalla HK. Integrated internal communications: a multidisciplinary perspective. Corporate Communications: An International Journal. 2005;10(4):302-14.

33.	Sinčić Ćorić D, Špoljarić A. The origins of internal communication and employer branding in marketing theories. Communication Management Review. 2021;6(01):30-45.

34.	Moroko L, Uncles MD. Characteristics of successful employer brands. Journal of Brand Management. 2008;16:151-75.

35.	Deepa R, Baral R. Relationship between integrated communication effectiveness and employee-based brand equity–mediating role of psychological contract fulfillment. Journal of Product & Brand Management. 2021;30(6):883-97.

36.	Leekha Chhabra N, Sharma S. Employer branding: strategy for improving employer attractiveness. International Journal of Organizational Analysis. 2014;22(1):48-60.

37.	Pérez-Pérez L, Berlanga I, Victoria JS. Internal communication and employer branding within a humanistic model–a case study of IKEA (Spain, 2019–2021). Corporate Communications: An International Journal. 2022(ahead-of-print):1-17.

38.	Itam U, Misra S, Anjum H. HRD indicators and branding practices: A viewpoint on the employer brand building process. European Journal of Training and Development. 2020;44(6/7):675-94.

39.	Pérez-Francisco DH, Duarte-Clíments G, del Rosario-Melián JM, Gómez-Salgado J, Romero-Martín M, Sánchez-Gómez MB, editors. Influence of workload on primary care nurses’ health and burnout, patients’ safety, and quality of care: Integrative review. Healthcare; 2020: Multidisciplinary Digital Publishing Institute.

40.	Nelke A. Impact of the COVID-19 pandemic on corporate employer branding. Technium Social Sciences Journal. 2021;16:388-93.

41.	Reed BN, Klutts AM, Mattingly TJ. A systematic review of leadership definitions, competencies, and assessment methods in pharmacy education. American journal of pharmaceutical education. 2019;83(9):1873-85.

42.	Zeesahn M, Qureshi TW, Bashir S, Ahmed U. Transformational Leadership and Corporate Reputation: Mediation Effects of Employer Branding. Journal of Management and Research. 2020;7(1):184-211.

43.	Yukl G. An evaluation of conceptual weaknesses in transformational and charismatic leadership theories. The Leadership Quarterly. 1999;10(2):285-305.

44.	Barrow S, Mosley R. The employer brand: Bringing the best of brand management to people at work. Chichester: John Wiley & Sons; 2011.

45.	Biswas M, Suar D. Antecedents and consequences of employer branding. Journal of Business Ethics. 2016;136(1):57-72.

46.	Dirani KM, Abadi M, Alizadeh A, Barhate B, Garza RC, Gunasekara N, et al. Leadership competencies and the essential role of human resource development in times of crisis: a response to Covid-19 pandemic. Human Resource Development International. 2020;23(4):380-94.

47.	Wooten LP, James EH. Linking crisis management and leadership competencies: The role of human resource development. Advances in Developing Human Resources. 2008;10(3):352-79.

48.	Glaser BG, Strauss AL, Strutzel E. The discovery of grounded theory; Strategies for qualitative research. London1968.

49.	Braun V, Clarke V. Conceptual and design thinking for thematic analysis. Qualitative Psychology. 2022;9(1):3.

50.	Cresswell J. Research Design: Qualitative2013.

51.	Shaheen M, Pradhan S. Sampling in Qualitative Research. Qualitative Techniques for Workplace Data Analysis. Pennsylvania: IGI Global; 2019. p. 25-51.

52.	Ritchie J, Lewis J, Elam G. Designing and selecting samples. In: Maruster L, editor. Qualitative research methods2003. p. 77-108.

53.	Etikan I, Musa SA, Alkassim RS. Comparison of convenience sampling and purposive sampling. American journal of theoretical and applied statistics. 2016;5(1):1-4.

54.	Palinkas LA, Horwitz SM, Green CA, Wisdom JP, Duan N, Hoagwood K. Purposeful sampling for qualitative data collection and analysis in mixed method implementation research. Administration and Policy in Mental Health and Mental Health Services Research. 2015;42:533-44.

55.	Patton MQ. Qualitative research and evaluation methods. 4 ed. Thousand Oaks, California: Sage; 2015.

56.	Monteiro B, Santos V, Reis I, Sampaio MC, Sousa B, Martinho F, et al. Employer branding applied to SMEs: A pioneering model proposal for attracting and retaining talent. Information. 2020;11(574):1-19.

57.	Biswas UN, Allard K, Pousette A, Härenstam A. Employer branding and attractive work. Understanding Attractive Work in a Globalized World: Springer; 2017. p. 27-35.

58.	Barriball KL, While A. Collecting data using a semi-structured interview: A discussion paper. Journal of Advanced Nursing. 1994;19(2):328-35.

59.	King N, Horrocks C. Remote interviewing. In: Horrocks C, editor. Qualitative Research. Los Angeles, California: Sage Publications; 2010. p. 79-102.

60.	Braun V, Clarke V. Using thematic analysis in psychology. Qualitative Research in Psychology. 2006;3(2):77-101.

61.	Braun V, Clarke V. What can "thematic analysis" offer health and wellbeing researchers? . Journal of Qualitative Studies on Health and Well-being. 2014;9(1):1947-52.

62.	O’Connor C, Joffe H. Intercoder reliability in qualitative research: debates and practical guidelines. International journal of qualitative methods. 2020;19:1609406919899220.

63.	Thomas DR. Feedback from research participants: are member checks useful in qualitative research? Qualitative research in psychology. 2017;14(1):23-41.

64.	Feng GC. Intercoder reliability indices: disuse, misuse, and abuse. Quality & Quantity. 2014;48:1803-15.

65.	Chen Y, Liu D, Tang G, Hogan TM. Workplace events and employee creativity: A multistudy field investigation. Personnel Psychology. 2021;74(2):211-36.

66.	Ouchi WG. Theory Z: How American business can meet the Japanese challenge. New York: Avon; 1982. 82-3 p.

67.	Slaughter JE, Zickar MJ, Highhouse S, Mohr DC. Personality Trait Inferences About Organizations: Development of a Measure and Assessment of Construct Validity. Journal of Applied Psychology. 2004;89(1):85-103.

68.	Lievens F, Highhouse S. The relation of instrumental and symbolic attributes to a company's attractiveness as an employer. Personnel Psychology. 2003;56(1):75-102.

69.	Nishii LH, Lepak DP, Schneider B. Employee attributions of the “why” of HR practices: Their effects on employee attitudes and behaviors, and customer satisfaction. Personnel Psychology. 2008;61(3):503-45.
