# Supplementary material for: Living the employer brand during a crisis? A qualitative study on internal employer branding in times of the COVID-19 pandemic
Source: PLoS One. 2024 May 13;19(5):e0303361. doi: 10.1371/journal.pone.0303361 (PMC11090342; doi:10.1371/journal.pone.0303361)
Supplement: Attachment — Submitted filename: 01_Response to Reviewers.docx [file pone.0303361.s007.docx]

Dear Editor,

Dear Dr. Georgantzis,

First, thank you for the opportunity to revise our manuscript “*Living the Employer Brand During a Crisis? A Qualitative Study on Internal Employer Branding in Times of the COVID-19 Pandemic”* again. We appreciated the many helpful comments and suggestions throughout this review process from you and the reviewers, which have enabled us to improve our manuscript.

As you will see in our reply to the reviewers’ comments and our revised paper, we carefully considered and answered all the comments. We believe this submission is an improvement from our submission and, thus, we are thankful for your and the reviewers’ comments and the opportunity to address them for a second time. A detailed account of all the changes made in reply to the comments is provided in the following pages. In the revised manuscript, titled ‘Revised Manuscript with Track Changes’, changes are highlighted by using red-colored text.

We would like to submit this revised version of our manuscript for publication in *PLOS ONE*.

We look forward to hearing from you.

Sincerely yours,

The authors

**EDITOR COMMENTS**

1. **Please review your reference list to ensure that it is complete and correct. If you have cited papers that have been retracted, please include the rationale for doing so in the manuscript text or remove these references and replace them with relevant current references. Any changes to the reference list should be mentioned in the rebuttal letter that accompanies your revised manuscript. If you need to cite a retracted article, indicate the article’s retracted status in the References list and also include a citation and full reference for the retraction notice.**

We appreciate the chance to revise the manuscript. After a thorough review, we found no retracted papers in the reference list. However, we have made some minor adjustments to ensure accuracy, such as correcting journal names, page numbers, issue numbers, and publication dates. These adjustments are indicated in red in the document titled 'Revised Manuscript with Track Changes'.

**REVIEWER 1 COMMENTS**

1. **Thank you for rigorously revising the manuscript. However, I still have one question regarding the data analysis. I see that you have followed my recommendations for reanalyzing with NVivo and checking the Inter-Rater Reliability. However, I found that you mentioned "*we have analyzed the transcripts in NVivo. Afterwards, to ensure the reliability of our coding, we conducted an intercoder reliability assessment. Intercoder reliability assesses the agreement among multiple researchers when assigning codes to themes. Following the recommendations of O’Connor and Joffe [62] and Feng [64], we opted for Krippendorf's alpha (Kalpha) as our measure of intercoder reliability. The Kalpha, calculated in SPSS, yielded substantial agreement. This serves as validation for the coding process and demonstrates that the interpretations and assigned codes maintain consistency across multiple researchers, thereby enhancing the overall reliability and credibility of the study.*" I am confused on how you said that you utilized NVivo (a qualitative analysis software) but producing the alpha numbers from SPSS (a quantitative analysis software). Is there anything that I am missing here?**

Thank you for recognizing the thorough revision of the manuscript. To address this discrepancy between utilizing NVivo for qualitative analysis and generating kalpha in SPSS, we would like to clarify our methodology further. While we did analyze the interviews using NVivo for the initial coding process and thematic analysis, we encountered challenges with NVivo's functionality, including frequent crashing, which jeopardized our progress. Consequently, to ensure the reliability of our coding, we opted for a manual intercoder reliability assessment. In line with the recommendation by O'Connor and Joffe (2020), who suggested the possibility of exporting qualitative data from a qualitative platform to a statistical software package (e.g., SPSS) for reliability assessment, we followed their guidelines in assessing intercoder reliability. This involved randomizing quotes into an Excel file and engaging another researcher to assign codes based on their interpretation, guided by the theoretical framework. Subsequently, we cross-validated these assignments manually and compiled the results in an Excel spreadsheet. Afterward, we opted to utilize SPSS to calculate Kalpha as a measure of intercoder reliability. In summary, while NVivo was utilized for the initial coding, the challenges we faced necessitated a manual approach for intercoder reliability assessment, with SPSS being used for the calculation of Kalpha.

**REVIEWER 2 COMMENTS**

1. **I would like to express my appreciation for the authors' extra efforts to meet the reviewers' concerns. The additional data collection (6 employee member checks) and further analysis with a focus on context of warm versus competence-oriented employer brand brings more nuance and richness to the conclusions. It also sharpens the contribution as internal employer branding study and not a leadership study during crisis periods.**

Thank you for acknowledging our efforts to address the suggestions from you and the reviewer team. We are pleased that the additional data collection and the subsequent analysis, which focuses on the warm versus competence-oriented context of employer branding, have contributed depth and nuance to our conclusions.

2. **The choice on internal communication and leadership as focal touchpoints in the internal branding strategies is communicated clearly now. I can fallow this choice, but I would also highlight in limitations section (where you shortly link in line 1016 to Instrumental-Symbolic framework for other touchpoints or strategies) that based on first five interviews and prior focus in literature this choice was made on these two touchpoints with the limitation of potentially having overlooked other important actions for internal employer branding strategies during crisis, most likely I assume for more competence oriented employer brands. I would also like to invite the authors to write consistently on internal communication and leadership as touchpoints of the internal employer branding strategies to avoid or rather minimize the perception that this is a study on leadership rather than on internal employer branding choices and strategies. For example, in starting lines of Discussion line 856-861 we read “This study aimed to acquire a better understanding of how HR managers perceived and experienced internal employer branding, internal communication, and leadership during a disruptive event.” This reads too much as a study on internal employer banding and on or rather on leadership. Might seem like a detail but prevents from misinterpreting the goal of the study on strategic approaches of HR managers to bring alive the desired internal employer brand along unstable times.**

Thank you for your feedback. We have adjusted the potential oversight of other important actions for internal employer branding strategies during crises. We have included following sentences in the manuscript:

“*Different theoretical frameworks, such as Instrumental-Symbolic Framework [27], could be used to incorporate a deeper understanding of how crisis events impact diverse employer brand orientations. Based on initial interviews and considering past literature, the decision was made to prioritize internal communication and leadership as touchpoints. However, we may have overlooked other important actions for internal employer strategies during times of crisis. As such, future research could also delve into (other) mechanisms or touchpoints through which organizations strategically align their internal branding efforts in response to crises, shedding light on the dynamic interplay between external uncertainties and internal organizational dynamics*.” (See page 42, lines 1024-1036)

Furthermore, we have taken care to consistently emphasize internal communication and leadership as touchpoints of the internal employer branding strategies, thereby minimizing the perception that our study primarily focuses on leadership rather than on internal employer branding choices and strategies. For instance, we have revised the introductory lines of the discussion section (lines 859-861) to better align with the overarching goal of our study, which is to understand the strategic approaches of HR managers in nurturing the desired internal employer brand during times of instability.

3. **In abstract it reads as if adopting warmth in the communication is the main result on internal employer branding strategy "adopting a warm communication style emerges as a facilitator in conveying the employer brand,” I would be careful as this message might conflict at first sight with the context specific approaches of more warmth and more competence-oriented employer brands despite the same challenges they faced during crisis. I do believe that the finding that despite organizational/operational constraints/risks the focus and energy is devoted to the experienced internal employer brand in crisis can benefit from being emphasized more boldly in abstract (it is also as an answer to the paradox the authors describe in introduction on immediate operational problems/needs and the internal brand attention to safeguard long term reputation and retention of current employees).**

Thank you for your insightful feedback. We appreciate your attention to the clarity of our abstract. We understand your concern regarding the potential misinterpretation of our main result as solely focusing on warmth in communication, which may conflict with the diverse approaches employed by different employer brands, particularly those emphasizing competence. As such, we have revised the abstract. Following paragraph was altered in the manuscript:

*“Employer branding has emerged as a strategic imperative in the quest for talent. However, existing research has predominantly explored stable periods, overlooking the possible transformative impact of crises and the crucial role that HR managers play in crafting internal employer branding strategies. As such, this research addresses this by scrutinizing internal employer branding during the COVID-19 pandemic. Conducting in-depth interviews with 37 Belgian HR managers, we delve into the perceived challenges and opportunities that the COVID-19 crisis presented with respect to internal employer branding and its touchpoints—internal communication and leadership. A subsequent member and employee check with six HR managers and six employees validated our findings. The results unveiled organizations' heightened concern for employer branding during crises, emphasizing the strategic reflection invested. Remarkably, despite facing organizational/operational constraints/risks imposed by the crisis, the attention and efforts remain steadfastly centered on the experienced internal employer brand in crisis situations. Additionally, a contextual analysis suggests that various employer brand types face similar challenges in crises, however, the employer brand serves as a defining factor that shapes how an organization responds to both external uncertainties and internal dynamics brought about by the crisis. This study contributes to a nuanced understanding of internal employer branding dynamics during crises, shedding light on the strategic considerations of HR managers.”* (See page 2, lines 26-43)

4. **An interesting finding is that employees do not per se interpret the internal branding efforts as such but rather as additional check-in time of their supervisors (see line 853). This might point to being careful by relying on employees’ views on perceived internal employer branding efforts as for them leadership style might be at play and hence be a confounder. It is of course a challenge to distinguish a leadership style in general from embracing the internal employer brand and value proposition in how you embody your leadership as supervisor. A critical reflection on this distinction might further help set apart your study as an internal employer branding study with leadership as touchpoint and a study on leadership styles in crisis periods.**

Thank you for highlighting this observation. We acknowledge that employees may interpret internal branding efforts differently than intended, perceiving them as additional interactions with their supervisors rather than specific employer branding initiatives. This insight underscores the importance of considering leadership style as a potential confounding factor in our study. We have taken your feedback into account and revised our paragraph, accordingly, reflecting on the distinction between internal employer branding and leadership styles during crisis periods. As such, we included in our manuscript the following paragraphs in the discussion:

“*Despite HR managers and the literature emphasizing the importance of supervisors in transferring the employer brand to employees, the participants in the employee check did not particularly perceive this. They rather perceived this as additional check-in time with their supervisor. This could suggest the need for caution when depending on employees' perspectives regarding employer branding efforts, as additional factors like leadership styles may play a role in how employees assess internal employer branding initiatives. Moreover, this incongruence between HR expectations and employee perceptions warrants further investigation. Future investigation may be required to explore the precise factors underlying this discrepancy, which could potentially stem from leadership style or the dynamics of supervisor-employee interactions. Investigating how various leadership styles, such as authoritarian, democratic, or coaching leadership, influence employees' interpretations of internal branding efforts is essential. For example, under a coaching supervisor, frequent check-ins can reinforce the perception of internal employer branding efforts by fostering supportive and collaborative interactions especially during challenging periods. Conversely, under an authoritarian supervisor, the intensified control and directive nature of conversations may play a role in how employees perceive the internal employer brand initiatives. Understanding these dynamics can provide valuable insights into optimizing the role of supervisors in internal employer branding and bridging the gap between managerial expectations and employee experiences.*” (see page 43, lines 1028-1046)

5. **Hope these final remarks may further support the authors in having a meaningful publication in the employer branding domain.**

Thank you for your encouraging final remarks. We appreciate your support in ensuring that our publication in the employer branding domain is meaningful.
